# Three new yeast species from flowers of *Camellia sinensis* var. *assamica* collected in Northern Thailand and their tannin tolerance characterization

**DOI:** 10.3389/fmicb.2023.1043430

**Published:** 2023-02-16

**Authors:** Apinun Kanpiengjai, Pratthana Kodchasee, Kridsada Unban, Jaturong Kumla, Saisamorn Lumyong, Pannida Khunnamwong, Dipayan Sarkar, Kalidas Shetty, Chartchai Khanongnuch

**Affiliations:** ^1^Division of Biochemistry and Biochemical Innovation, Department of Chemistry, Faculty of Science, Chiang Mai University, Chiang Mai, Thailand; ^2^Research Center for Multidisciplinary Approaches to Miang, Chiang Mai University, Chiang Mai, Thailand; ^3^Division of Biotechnology, Faculty of Agro-Industry, Chiang Mai University, Chiang Mai, Thailand; ^4^Division of Food Science and Technology, Faculty of Agro-Industry, Chiang Mai University, Chiang Mai, Thailand; ^5^Division of Microbiology, Department of Biology, Faculty of Science, Chiang Mai University, Chiang Mai, Thailand; ^6^Research Center of Microbial Diversity and Sustainable Utilization, Faculty of Science, Chiang Mai University, Chiang Mai, Thailand; ^7^Department of Microbiology, Faculty of Science, Kasetsart University, Bangkok, Thailand; ^8^Biodiversity Center Kasetsart University (BDCKU), Bangkok, Thailand; ^9^Global Institute of Food Security and International Agriculture (GIFSIA), Department of Plant Sciences, North Dakota State University, Fargo, ND, United States

**Keywords:** Miang, tannin-tolerance, tannase, taxonomy, tea flower, yeast

## Abstract

Our recent research study focused on Miang fermentation revealed that tannin-tolerant yeasts and bacteria play vital roles in the Miang production process. A high proportion of yeast species are associated with plants, insects, or both, and nectar is one of the unexplored sources of yeast biodiversity. Therefore, this study aimed to isolate and identify yeasts of tea flowers of *Camellia sinensis* var. *assamica* and to investigate their tannin tolerance, which is a property essential to Miang production processes. A total of 82 yeasts were recovered from a total of 53 flower samples in Northern Thailand. It was found that two and eight yeast strains were distinct from all other known species within the genera *Metschnikowia* and *Wickerhamiella*, respectively. These yeast strains were described as three new species, namely, *Metschnikowia lannaensis*, *Wickerhamiella camelliae*, and *W. thailandensis*. The identification of these species was based on phenotypic (morphological, biochemical, and physiological characteristics) and phylogenetic analyses of a combination of the internal transcribed spacer (ITS) regions and the D1/D2 domains of the large subunit (LSU) ribosomal RNA gene. The yeast diversity in tea flowers acquired from Chiang Mai, Lampang, and Nan provinces had a positive correlation with those acquired from Phayao, Chiang Rai, and Phrae, respectively. *Wickerhamiella azyma*, *Candida leandrae*, and *W. thailandensis* were the species uniquely found in tea flowers collected from Nan and Phrae, Chiang Mai, and Lampang provinces, respectively. Some of the tannin-tolerant and/or tannase-producing yeasts were associated with yeasts in the commercial Miang process and those found during Miang production, i.e., *C. tropicalis*, *Hyphopichia burtonii*, *Meyerozyma caribbica*, *Pichia manshurica*, *C. orthopsilosis*, *Cyberlindnera fabianii*, *Hanseniaspora uvarum*, and *Wickerhamomyces anomalus*. In conclusion, these studies suggest that floral nectar could support the formation of yeast communities that are beneficial for Miang production.

## 1. Introduction

*Camellia sinensis* var. *assamica* is one of the important materials for processing various tea products, specifically oolong, black, and Pu-erh (Wang et al., 2013). It is suggested by some researchers that *C. sinensis* var. *assamica* is the variant of *C. sinensis* var. *sinensis*, another major variety grown for tea production to date (Zhang et al., 2019). Miang is a fermented food product traditionally produced from the leaves of *C*. *sinensis* var. *assamica* in the mountainous areas of Northern Thailand. It is different from other tea products, and it is known as eating tea or chewing tea. Miang also has long deep-rooted social and cultural integration and relevance with the people of Northern Thailand (Khanongnuch et al., 2017). In our previous studies, several synergistic interactions were observed among bacteria, yeasts, and filamentous fungi in Miang, which was associated with the release of several bioactive compounds particularly gallated catechins and gallated epicatechin (Unban et al., 2019, 2020a; Kodchasee et al., 2021). Most associated microorganisms, especially bacteria and yeasts, exhibited specific characteristics in terms of tannin tolerance and tannase production (Kanpiengjai et al., 2016; Chaikaew et al., 2017). Production of lignocellulose-degrading enzymes and tannase by tannin-tolerant microorganisms is believed to be essential for the release of useful therapeutic phenolic compounds, specifically catechins, tannins, and gallic acid (Unban et al., 2020b). To identify the deep-rooted microbial environment during tea leaf fermentation, bacterial and fungal diversities were determined (Unban et al., 2020a; Kodchasee et al., 2021). Based on the previous results, it was clearly demonstrated that tannin-tolerant ability of microorganisms is aligned with their taxonomy, specifically at family, genus, and species levels and is one of the key criteria for evaluating the evolution of microorganisms associated with Miang fermentation (Kanpiengjai et al., 2016; Chaikaew et al., 2017; Unban et al., 2020b). Plants offer a great variety of favorable microenvironments specifically for yeast growth. Unique yeast communities have been associated with the rhizosphere, the phylloplane, plant exudates, plant necrotic tissues, fruits, and flowers (De Vega et al., 2017). In a previous study, several yeasts, including *Aureobasidium melanogenum*, *Kazachstania aquatica*, *Saturnispora diversa*, *Saturnispora sekii*, *Schwanniomyces pseudopolymorphus*, *Wickerhamomyces anomalus*, *Apiotrichum scarabaeorum*, *Curvibasidium pallidicorallinum*, *Papiliotrema laurentii*, *Rhodosporidiobolus ruineniae*, *Trichosporon asahii*, and *T*. *coremiiforme*, were discovered from the soil of Assam tea (Kumla et al., 2020), but their taxonomic alignment is not associated with yeasts found in Miang. The current study has revealed that nectar-inhabiting microorganisms are likely to interact with plants and pollinators. Yeasts, commonly present in nectar, can modify a variety of nectar traits that are important for pollinator attraction (Schaeffer et al., 2017). Therefore, the yeast community in Assam tea flowers may act as a reservoir of yeasts that are associated with the Miang production process as well as unique Miang products from each production area. Therefore, this study aimed to investigate the yeast ecology of Assam tea flowers collected from six provinces in upper Northern Thailand by using the culture-dependent method and to characterize the tannin tolerance capability of the isolated yeasts. The study insights can explain the association between flower yeasts and Miang fermentation. To our knowledge, this is the first report to describe the isolation and molecular-based identification of yeasts associated with tea flowers.

## 2. Materials and methods

### 2.1. Chemical and media

Tannin (gallotannin) was purchased from LOBA Chemie (Mumbai, India), and methyl gallate was purchased from Sigma (Steinheim, Germany). Ingredients such as peptone, yeast extract, malt extract, and carbon sources such as glucose, galactose, sucrose, maltose, lactose, raffinose, and trehalose were purchased from HiMedia (Nashik, India). The genomic DNA of yeast was extracted using a Wizard^®^ Genomic DNA purification kit (Promega Corp., Madison, WI, United States). Nucleic acid amplifications were performed using TaKaRa Ex Taq^®^ (Shiga, Japan). Other reagents and solvents were of analytical grade.

### 2.2. Sampling site and sample collection

**Figure 1:**
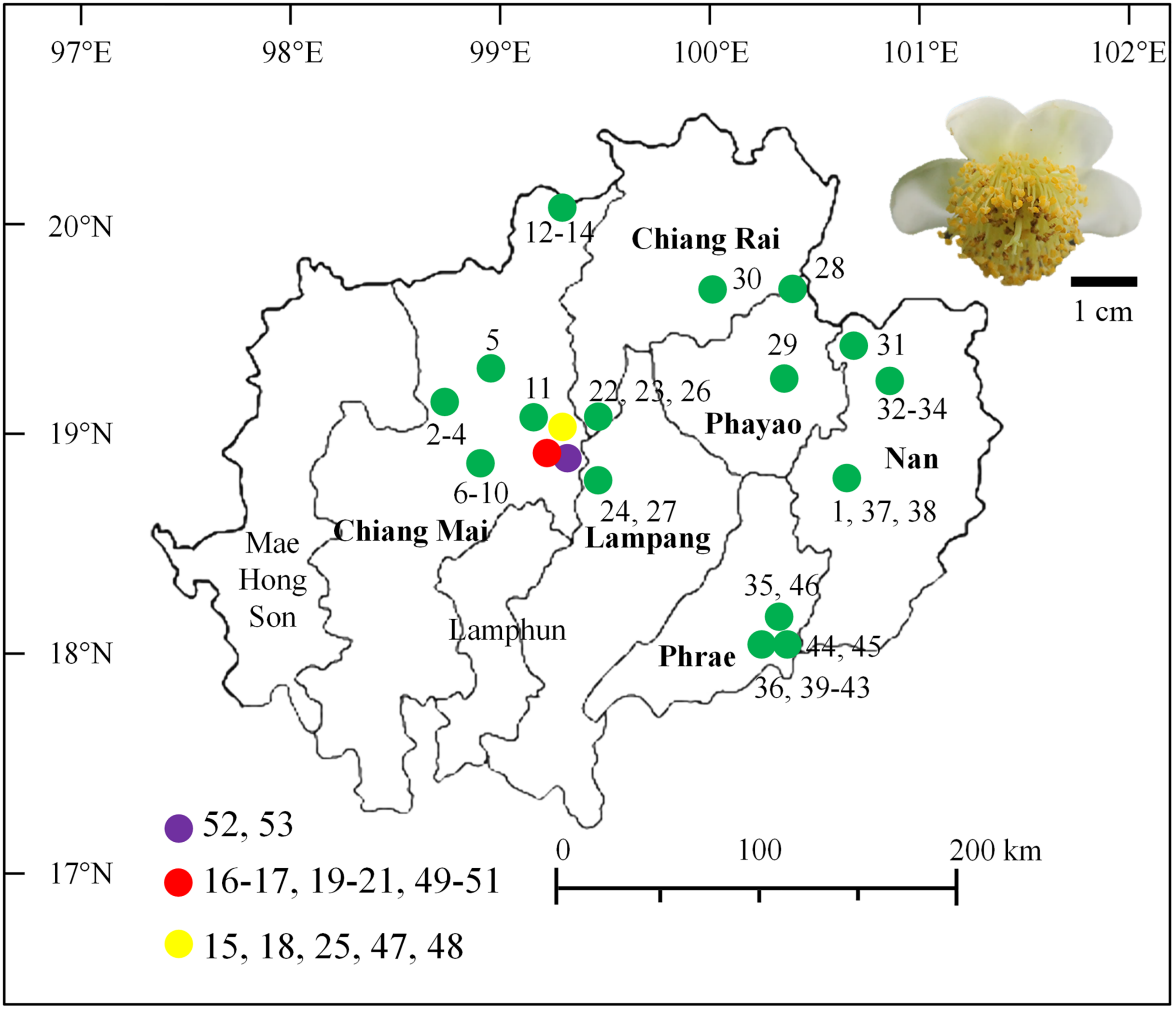
Map of the area indicates the tea flower sampling sites in Northern Thailand and a photo of a tea flower.

Assam tea flower samples were directly collected from 53 sites that were located within 19 tea plantations used for Miang production from December 2015 to May 2016. These locations covered all regions of the Miang production area, which include six provinces of upper Northern Thailand, namely, Chiang Mai, Chiang Rai, Phrae, Nan, Lampang, and Phayao (Figure 1), where they were recorded to be the Miang production area in previous studies (Khanongnuch et al., 2017). The main tea plantations for Miang production are in Chiang Mai (28 sites in eight locations), Phrae (10 sites in three locations), Nan (seven sites in three locations), Chiang Rai (four sites in two locations), Phayao (two sites in two locations), and Lampang (one location). Each tea flower sample was aseptically collected from each sampling site, put in a sterile plastic bag, date recorded, stored on ice, and delivered to the laboratory within 24 h prior to the sample preparation and further yeast isolation. The sampling site, location, and date of the collection were recorded and given code numbers (Supplementary Table S1).

### 2.3. Isolation and purification of yeasts

Approximately 1 g of the tea flower sample was homogenized in 10 mL of sterile 0.85% NaCl as a diluent using a Masticator^®^ homogenizer blender (Barcelona, Spain) for 5 min. After that, the homogenate was serially diluted to 10^0^–10^5^ with the diluent, and 0.1 mL of each dilution was plated on yeast–malt extract agar (YMA) (1% yeast extract, 2% peptone, 2% glucose, and 1.5% agar) supplemented with 100 mg/L of chloramphenicol and then incubated at 30°C for 48 h. Yeast colonies with distinct morphologies were isolated and streaked on the same medium without the supplementation of chloramphenicol for purification (Khunnamwong et al., 2022). For storage, a single colony of each yeast isolate was inoculated in yeast–malt extract broth (YMB) (1% yeast extract, 2% peptone, and 2% glucose) and incubated at 30°C on a 150-rpm rotary shaker for 24 h. The culture was mixed with 30% (v/v) glycerol to make a final concentration of 15% before being stored at −80°C.

### 2.4. Yeast identification

#### 2.4.1. Molecular identification

Amplification of the D1/D2 domain of the large subunit (LSU) rRNA gene or the internal transcribed spacer (ITS) region was performed by a standard polymerase chain reaction (PCR) using genomic DNA of each yeast as a template with forward primer NL1 (5′-GCATATCAATAAGCGGGGAAAAG-3′) and reverse primer NL4 (5′-GGTCCGTGTTTCAAGACGG-3′), and forward primer ITS1 (5′-TCCGTAGGTGAACCTGCGG-3′) and reverse primer ITS4 (5′-TCCTCCGCTTATTGATATGC-3′), respectively. Amplicons obtained from PCR were purified and sequenced by a sequencing service provider (1st BASE Ptd Ltd., Singapore).

Analysis of the ITS and D1/D2 sequences was conducted by similarity searches using the BLAST program available at NCBI.^1^ The yeast strains showing nucleotide substitutions of greater than 1% in the D1/D2 domain of the LSU rRNA gene are usually considered different species (Kurtzman and Robnett, 1997) and were selected for further phylogenetic analysis and phenotypic characterization. The holotypes of new species were preserved in a metabolically inactive state in the culture collection of the Division of Biotechnology, Faculty of Agro-Industry, Chiang Mai University, Chiang Mai, Thailand. The ex-type cultures were deposited in a metabolically inactive state at the culture collection of the Sustainable Development of Biological Resources, Faculty of Science, Chiang Mai University (SDBR-CMU), Chiang Mai, Thailand.

#### 2.4.2. Phylogenetic analysis of new yeast species

The sequences from this study and those obtained from previous studies together with sequences downloaded from the nucleotide GenBank database were selected and provided for phylogenetic analysis. Multiple sequence alignment was performed with MUSCLE (Edgar, 2004) and manually checked using BioEdit v. 6.0.7 (Hall, 1999). Phylogenetic analysis was performed based on the combined dataset of ITS and D1/D2 sequences. A phylogenetic tree was constructed using maximum likelihood (ML) and Bayesian inference (BI) methods. The best substitution models for ML and BI analyses were estimated by the Akaike information criterion (AIC) in jModelTest 2.1.10 (Darriba et al., 2012). ML analysis was carried out on RAxML v7.0.3 under the GTRCAT model with 25 categories and 1,000 bootstrap (BS) replications (Felsenstein, 1985; Stamatakis, 2006) *via* the online portal CIPRES Science Gateway v. 3.3 (Miller et al., 2010). BI analysis was performed by MrBayes v3.2.6 (Ronquist and Huelsenbeck, 2003). For the BI analysis, six simultaneous Markov chains were run for one million generations with random initial trees, wherein every 1,000 generations were sampled. A burn-in phase was employed to discard the first 2,000 of the trees, while the remaining trees were used to construct the 50% majority-rule consensus phylogram with calculated Bayesian posterior probabilities (PPs). Tree topologies were visualized in FigTree v1.4.0 (Rambaut, 2016).

#### 2.4.3. Morphology characterization of new yeast species

The morphological characteristics of expected new yeast species were determined according to the standard methods described by Kurtzman et al. (2011), De Vega et al. (2017), and Shibayama et al. (2020). Colony formation was observed on YMA after incubation at 25°C for 3 days. The formation of pseudohyphae and true hyphae was investigated by cultivation on potato dextrose agar (PDA) (20% potato infusion, 2% glucose, and 1.5% agar) in slide culture at 25°C for up to 4 weeks. Ascospore formation was investigated for individual strains and strain pairs on PDA, corn meal agar, 5% malt extract agar (5% malt extract and 1.5% agar), yeast carbon base agar supplemented with 0.01% yeast extract (YCBY), Fowell’s acetate agar, yeast extract-peptone dextrose (YPD) agar (1% yeast extract, 2% peptone, 2% glucose, and 1.5% agar), glucose yeast (GY) agar (1% glucose, 1% yeast extract, 2% agar), and YMA at 25°C for 4 weeks. Macromorphological characteristics were examined under a light microscope (Nikon Eclipse Ni U, Tokyo, Japan). Size data of the anatomical structure such as cells, pseudohyphae, asci, and ascospores were based on at least 50 measurements of each structure using the Tarosoft^®^ Image Frame Work program.

#### 2.4.4. Biochemical and physiological characterization of new yeast species

All strains of the new yeast species were characterized biochemically and physiologically, according to the standard methods described by Kurtzman et al. (2011). Fermentation of carbohydrates was carried out in a liquid medium using Durham fermentation tubes. Carbon source and nitrogen source assimilation tests were carried out in a liquid medium, and starved inocula were used in the nitrogen assimilation test. Cycloheximide resistance was also performed in a liquid medium, while urea hydrolysis was conducted on an agar slant. Acid production and the diazonium blue B (DBB) reaction were investigated on the solid media in Petri dishes. The effect of temperature on yeast growth was investigated using YMA at various temperatures ranging from 15 to 37°C. The ability to grow in media of high osmotic pressure was performed using 50 and 60% glucose agar, and 10 and 16% sodium chloride plus 5% glucose medium.

### 2.5. Determination of yeast diversity isolated from tea flowers

In the yeast isolation step, each tea flower sample was collected from each sampling site. The number of isolated yeasts from each sample was recorded, calculated, and further expressed in terms of the number of yeasts per sample. After the yeast identification step, the number of samples found for each species was recorded. The frequency of each species was calculated by the number of samples that contained certain species divided by the total number of tea flower samples. Principal component analyses (PCA) based on the relative abundance of yeast species in tea flower samples and provinces were performed using GraphPad Prism9 software version 9.4.1 (GraphPad Software, San Diego, CA, United States).

### 2.6. Tannin-tolerant test and detection of tannase

Tannin-tolerant test was evaluated by inoculating a single yeast colony on the YMA plate with and without the supplementation of 10, 20, 30, 40, and 50 g/L tannic acid according to the method described in a previous study (Kanpiengjai et al., 2016). Growth of yeasts was observed after incubation at 30°C for 24 to 72 h. Principal component analyses (PCA) based on the relative abundance of tannin-tolerant yeast species in tea flower samples and provinces were performed.

Detection of tannase was also performed by a visual reading method (Osawa and Walsh, 1993). Briefly, fresh cultures on YMA containing 10 g/L tannic acid were harvested and suspended in 750 μL of substrate medium containing 33 mM NaH_2_PO_4_ and 20 mM methyl gallate for the purposes of preparing a dense suspension. The substrate medium was incubated aerobically at 30°C for 24 h. The mixture was later alkalinized by adding an equal amount of a saturated NaHCO_3_ solution (pH 8.6) and then incubating it at 25°C for 1 h. This suspension was centrifuged to remove any cells, and the absorbance of the supernatant was measured at 440 nm. The supernatant changed color from green to brown. An absorbance value of higher than 0.5 was considered a positive indication of tannase activity.

## 3. Results

### 3.1. Yeast isolation and identification

Based on colony and shape, a total of 82 yeast isolates were generated from 47 out of 53 tea flower samples. The number of yeast strains was dependent on the number of sampling sites derived from each province; thus, Chiang Mai was the major source of the isolated yeasts. The number of yeast isolates per sample was between 0 and 6 isolates per sample (Supplementary Table S1). Two samples possessed the highest number of yeast isolates with 4–6 isolates per sample. They included tea flowers from Na Rai Luang subdistrict, Nan (FLA31), and Longkhod subdistrict, Chiang Mai (FLA11), while others were recorded to have the ratio of yeast species and site in a range of 0 to 3 isolates per site.

Initially, all yeasts were identified using sequence analysis of the D1/D2 domain of the LSU rRNA gene. The sequence of the D1/D2 domain was aligned with the related type strains deposited in NCBI. For each species, the sequence of the D1/D2 domain that shared more than 98% with those of the closest species is shown in Table 1. In total, 72 yeast strains were identified within 39 known yeast species, while another 10 yeast strains were representative of three undescribed species in the *Wickerhamiella* (strains FLA5.4, FLA13.1, FLA23.2, FLA46.4, FLA4.2, FLA24.1, FLA27.3, and FLA30.2) and *Metschnikowia* (FLA8.2 and FLA27.1) clade (Supplementary S2). In addition, each strain of the three undescribed species was isolated from a different location in the tea plantation area.

**Table 1:** Identification of yeast strains isolated from tea flower and their GenBank accession numbers of the D1/D2 domain of LSU rRNA gene.

Isolate	Accession No.	CBS type species	Identification/Similarity (%)	Location	Sites (subdistrict/district)	Province	No. of sample found for each species	% Frequency of occurrence
FLA4.1	MW602315	*Candida leandrae* CBS 9735	*C*. *leandrae*/99.80	19°07′00.3″N 98°43′35.0″E	Site 3: Papae/ Mae Taeng	Chiang Mai	9	10.6
FLA5.1	MW602316	*Candida leandrae* CBS 9735	*C*. *leandrae*/99.80	19°44′01.9″N 98°54′04.1″E	Site 1: Mae Na/ Chiang Dao	Chiang Mai		
FLA7.1	MW602319	*Candida leandrae* CBS 9735	*C*. *leandrae*/99.80	18°50′29.3″N 98°54′15.9″E	Site 2: Suthep/ Muang	Chiang Mai		
FLA8.1	MW602320	*Candida leandrae* CBS 9735	*C*. *leandrae*/99.80	18°50′29.3″N 98°54′15.9″E	Site 3: Suthep/ Muang	Chiang Mai		
FLA9.1	MW602322	*Candida leandrae* CBS 9735	*C*. *leandrae*/99.80	18°50′29.3″N 98°54′15.9″E	Site 4: Suthep/ Muang	Chiang Mai		
FLA15.1	MW602331	*Candida leandrae* CBS 9735	*C*. *leandrae*/99.80	18°57′41.2″N 99°14′06.9″E	Site 1: Pa Miang/ Doi Saket	Chiang Mai		
FLA16.1	MW602332	*Candida leandrae* CBS 9735	*C*. *leandrae*/99.80	18°57′46.2″N 99°21′22.3″E	Site 1: Thepsadet/ Doi Saket	Chiang Mai		
FLA19.1	MW602335	*Candida leandrae* CBS 9735	*C*. *leandrae*/99.80	18°57′46.2″N 99°21′22.3″E	Site 3: Thepsadet/ Doi Saket	Chiang Mai		
FLA28.2	MW602340	*Candida leandrae* CBS 9735	*C*. *leandrae*/99.80	19°35′19.1″N 100°22′30.0″E	Site 1: Phu Sang/ Chiang Kham	Phayao		
FLA44.2	MW602372	*Candida orthopsilosis* CBS 10744	*C*. *orthopsilosis*/100.00	17°59′59.2″N 100°15′19.7″E	Site 1: Cho Hae/ Muang	Phrae	2	2.4
FLA46.2	MW602376	*Candida orthopsilosis* CBS 10744	*C*. *orthopsilosis*/100.00	18°07′33.4″N 100°18′57.5″E	Site 2: Suan Khuen/ Muang	Phrae		
FLA40.1	MW602364	*Clavispora pseudohaemulonis* CBS 10004	*Cl*. *pseudohaemulonis*/99.21	18°07′02.0″N 100°17′50.9″E	Site 3: Pa Daeng/ Muang	Phrae	1	1.2
FLA39.2	MW602345	*Cyberlindnera fabianii* CBS 5640	*Cy*. *fabianii*/100.00	18°07′02.0″N 100°17′50.9″E	Site 2: Pa Daeng/ Muang	Phrae	1	1.2
FLA32.2	MW602349	*Debaryomyces fabryi* CBS 789	*D*. *fabryi*/100.00	19°14′41.0″N 100°59′52.0″E	Site 1: Sakad/ Pua	Nan	1	1.2
FLA31.1	MW602342	*Debaryomyces nepalensis* CBS 7761	*D*. *natalensis*/100.00	19°23′06.9″N 100°41′46.7″E	Site 1: NaRai Luang/ Song Khwae	Nan	2	2.4
FLA46.3	MW602377	*Debaryomyces nepalensis* CBS 7761	*D*. *nepalensis*/100.00	18°07′33.4″N 100°18′57.5″E	Site 2: Suan Khuen/ Muang	Phrae		
FLA34	MW602352	*Debaryomyces subglobosus* CBS 792	*D*. *subglobosus*/100.00	19°14′41.0″N 100°59′52.0″E	Site 3: Sakad/ Pua	Nan	1	1.2
FLA33	MW602351	*Diutina catenulata* CBS 565	*Di*. *catenulata*/100.00	19°14′41.0″N 100°59′52.0″E	Site 2: Sakad/ Pua	Nan	1	1.2
FLA9.2	MW602323	*Hanseniaspora uvarum* CBS 104	*H*. *uvarum*/99.65	18°50′29.3″N 98°54′15.9″E	Site 4: Suthep/ Muang	Chiang Mai	1	1.2
FLA31.6	MW602346	*Hyphopichia buttonii* CBS 4571	*Hy*. *burtonii*/100.00	19°23′06.9″N 100°41′46.7″E	Site 1: NaRai Luang/ Song Khwae	Nan	1	1.2
FLA31.4	MW602344	*Kodamaea ohmeri* CBS 5367	*K*. *ohmeri*/100.00	19°23′06.9″N 100°41′46.7″E	Site 1: NaRai Luang/ Song Khwae	Nan	2	2.4
FLA46.1	MW602375	*Kodamaea ohmeri* CBS 5367	*K*. *ohmeri*/100.00	18°07′33.4″N 100°18′57.5″E	Site 2: Suan Khuen/ Muang	Phrae		
FLA11.1	MW602325	*Kodamaea restingae* CBS 8492	*K*. *restingae*/98.58	19°05′43.3″N 99°10′54.5″E	Site 1: Longkhod/ Phrao	Chiang Mai	1	1.2
FLA11.3	MW602327	*Kurtzmaniella quercitrusa* CBS 4412	*Ku*. *quercitrusa*/100.00	19°05′43.3″N 99°10′54.5″E	Site 1: Longkhod/ Phrao	Chiang Mai	3	3.5
FLA12.1	MW602330	*Kurtzmaniella quercitrusa* CBS 4412	*Ku*. *quercitrusa*/99.82	19°05′43.3″N 99°10′54.5″E	Site 1: Mae Ai/ Mai Ai	Chiang Mai		
FLA29.1	MW602341	*Kurtzmaniella quercitrusa* CBS 4412	*Ku*. *quercitrusa*/100.00	19°14′18.4″N 100°19′50.1″E	Site 1: Oi/ Pong	Phayao		
FLA47.1	MW602378	*Meryerozyma caribbica* CBS 9966	*Me. caribbica*/100.00	18°55′24.7″N 99°12′03.5″E	Site 4: Pa Miang/ Doi Saket	Chiang Mai	1	1.2
FLA22.1	MW602337	*Metschnikowia chrysomelidarum* CBS 9904	*M*. *chrysomelidarum*/99.58	19°05′16.8″N 99°22′29.6″E	Site 1: MaeChedi Mai/ Wiang Papao	Chiang Rai	1	1.2
FLA6.1	MW602318	*Metschnikowia hawaiiana* CBS 9146	*M. hawaiiana*/99.80	18°50′29.3″N 98°54′15.9″E	Site 1: Suthep/ Muang	Chiang Mai	3	3.5
FLA32.1	MW602348	*Metschnikowia hawaiiana* CBS 9146	*M. hawaiiana*/100.00	19°14′41.0″N 100°59′52.0″E	Site 1: Sakad/ Pua	Nan		
FLA43.1	MW602370	*Metschnikowia hawaiiana* CBS 9146	*M. hawaiiana*/100.00	18°07′02.0″N 100°17′50.9″E	Site 6: Pa Daeng/ Muang	Phrae		
FLA1.2	MW602309	*Metschnikowia koreensis* CBS 8854	*M*. *koreensis*/100.00	18°47′08.5″N 100°39′37.5″E	Site 1: Ruang/ Muang	Nan	2	2.4
FLA41.1	MW602367	*Metschnikowia koreensis* CBS 8854	*M*. *koreensis*/100.00	18°07′02.0″N 100°17′50.9″E	Site 4: Pa Daeng/ Muang	Phrae		
FLA31.2	MW602343	*Metschnikowia laotica* CBS 12961	*M*. *laotica*/99.20	19°23′06.9″N 100°41′46.7″E	Site 1: Na Rai Luang/ Song Khwae	Nan	1	1.2
FLA2.3	MW602312	*Metschnikowia rancensis* CBS 8174	*M*. *rancensis*/100.00	19°07′16.8″N 98°42′28.9″E	Site 1: Papae/ Mae Taeng	Chiang Mai	4	4.7
FLA18.1	MW602334	*Metschnikowia rancensis* CBS 8174	*M*. *rancensis*/99.80	18°57′42.3″N 99°14′04.9″E	Site 2: Pa Miang/ Doi Saket	Chiang Mai		
FLA22.2	MW602338	*Metschnikowia rancensis* CBS 8174	*M*. *rancensis*/99.80	19°05′16.8″N 99°22′29.6″E	Site 1: MaeChedi Mai/ Wiang Papao	Chiang Rai		
FLA38.1	MW602359	*Metschnikowia rancensis* CBS 8174	*M*. *rancensis*/99.80	18°47′08.5″N 100°39′37.5″E	Site 3: Ruang/ Muang	Nan		
FLA11.4	MW602328	*Nakazawaea ishiwadae* CBS 6022	*N*. *ishiwadae*/100.00	19°05′43.3″N 99°10′54.5″E	Site 1: Longkhod/ Phrao	Chiang Mai	1	1.2
FLA8.2	KY640629	*Metschinikowia hawiiana* CBS 9146	*Metschinikowia* sp./89.35	18°50′29.3″N 98°54′15.9″E	Site 3: Suthep/ Muang	Chiang Mai	2	2.4
FLA27.1	MW542582	*Metschinikowia hawiiana* CBS 9146	*Metschinikowia* sp./89.16	18°49′43.4″N 99°23′14.6″E	Site 2: Chae Son/ Muang Pan	Lampang		
FLA5.4	KY411895	*Wickerhamiella natalensis* CBS 14161	*Wickerhamiella* sp./96.95	19°44′01.9″N 98°54′04.1″E	Site 1: Mae Na/ Chiang Dao	Chiang Mai	4	4.7
FLA13.1	MW542579	*Wickerhamiella natalensis* CBS 14161	*Wickerhamiella* sp./96.59	20°05′09.9″N 99°17′11.0″E	Site 2: Mae Ai/ Mae Ai	Chiang Mai		
FLA23.2	MW542580	*Wickerhamiella natalensis* CBS 14161	*Wickerhamiella* sp./96.95	19°05′16.8″N 99°22′29.6″E	Site 2: MaeChedi Mai/ Wiang Papao	Chiang Rai		
FLA46.4	MW542585	*Wickerhamiella natalensis* CBS 14161	*Wickerhamiella* sp./96.95	18°07′33.4″N 100°18′57.5″E	Site 2: Suan Khuen/ Muang	Phrae		
FLA4.2	KY411893	*Wickerhamiella musiphila* CBS10697	*Wickerhamiella* sp./94.38	19°07′00.3″N 98°43′35.0″E	Site 3: Papae/ Mae Taeng	Chiang Mai	4	4.7
FLA24.1	MW542581	*Wickerhamiella musiphila* CBS 10697	*Wickerhamiella* sp./94.38	18°49′43.4″N 99°23′14.6″E	Site 1: Chae Son/ Muang Pan	Lampang		
FLA27.3	KY640634	*Wickerhamiella musiphila* CBS 10697	*Wickerhamiella* sp./94.57	18°49′43.4″N 99°23′14.6″E	Site 2: Chae Son/ Muang Pan	Lampang		
FLA30.2	MW542584	*Wickerhamiella musiphila* CBS 10697	*Wickerhamiella* sp./94.57	19°34′00.7″N 100°02′35.8″E	Site 1: Mae Loi/ Thoeng	Chiang Rai		
FLA39.3	MW602363	*Pichia manshurica* CBS 209	*P*. *manshurica*/100.00	18°07′02.0″N 100°17′50.9″E	Site 2: Pa Daeng/ Muang	Phrae	3	3.5
FLA40.3	MW602365	*Pichia manshurica* CBS 209	*P*. *manshurica*/100.00	18°07′02.0″N 100°17′50.9″E	Site 3: Pa Daeng/ Muang	Phrae		
FLA41.3	MW602368	*Pichia manshurica* CBS 209	*P*. *manshurica*/100.00	18°07′02.0″N 100°17′50.9″E	Site 4: Pa Daeng/ Muang	Phrae		
FLA48.1	MW602739	*Priceomyces melissophilus* CBS 6344	*Pr*. *melissophilus*/98.80	18°55′24.7″N 99°12′03.5″E	Site 5: Pa Miang/ Doi Saket	Chiang Mai	1	1.2
FLA42.1	MW602369	*Saccharomyces cerevisiae* CBS 5493	*S*. *cerevisiae*/100.00	18°07′02.0″N 100°17′50.9″E	Site 5: Pa Daeng/ Muang	Phrae	1	1.2
FLA17.1	MW602333	*Saccharomycopsis fodiens* CBS 8332	*Sc*. *fodiens*/100.00	18°55′15.8″N 99°19′49.0″E	Site 2: Thepsadet/ Doi Saket	Chiang Mai	1	1.2
FLA5.3	KY411894	*Suhomyces xylopsoci* CBS 6037	*Su*. *xylopsoci*/98.40	19°44′01.9″N 98°54′04.1″E	Site 1: Mae Na/ Chiang Dao	Chiang Mai	3	3.5
FLA44.1	MW602371	*Suhomyces xylopsoci* CBS 6037	*Su*. *xylopsoci*/98.57	17°59′59.2″N 100°15′19.7″E	Site 1: Cho Hae/ Muang	Phrae		
FLA45.1	MW602373	*Suhomyces xylopsoci* CBS 6037	*Su*. *xylopsoci*/98.57	17°59′59.2″N 100°15′19.7″E	Site 2: Cho Hae/ Muang	Phrae		
FLA1.3	MW602310	*Wickerhamiella azyma* CBS 6826	*W*. *azyma*/99.28	18°47′08.5″N 100°39′37.5″E	Site 1: Ruang/ Muang	Nan	8	9.4
FLA8.3	MW602321	*Wickerhamiella azyma* CBS 6826	*W*. *azyma*/99.28	18°50′29.3″N 98°54′15.9″E	Site 3: Suthep/ Muang	Chiang Mai		
FLA20.1	MW602336	*Wickerhamiella azyma* CBS 6826	*W*. *azyma*/99.28	18°55′18.7″N 99°19′54.4″E	Site 4: Thepsadet/ Doi Saket	Chiang Mai		
FLA35.2	MW602354	*Wickerhamiella azyma* CBS 6826	*W*. *azyma*/99.28	18°07′33.4″N 100°18′57.5″E	Site 1: Suan Khuen/ Muang	Phrae		
FLA36.3	MW602357	*Wickerhamiella azyma* CBS 6826	*W*. *azyma*/99.28	18°07′02.0″N 100°17′50.9″E	Site 1: Pa Daeng/ Muang	Phrae		
FLA37.1	MW602358	*Wickerhamiella azyma* CBS 6826	*W*. *azyma*/99.64	18°47′08.5″N 100°39′37.5″E	Site 2: Ruang/ Muang	Nan		
FLA38.2	MW602360	*Wickerhamiella azyma* CBS 6826	*W*. *azyma*/99.28	18°47′08.5″N 100°39′37.5″E	Site 3: Ruang/ Muang	Nan		
FLA40.4	MW602366	*Wickerhamiella azyma* CBS 6826	*W*. *azyma*/99.27	18°07′02.0″N 100°17′50.9″E	Site 3: Pa Daeng/ Muang	Phrae		
FLA39.1	MW602361	*Wickerhamomyces anomalus* CBS 5759	*Wc*. *anomalus*/100.00	18°07′02.0″N 100°17′50.9″E	Site 2: Pa Daeng/ Muang	Phrae	1	1.2
FLA11.2	MW602326	*Wickerhamomyces ciferrii* CBS 111	*Wc*. *ciferrii*/100.00	19°05′43.3″N 99°10′54.5″E	Site 1: Longkhod/ Phrao	Chiang Mai	1	1.2
FLA45.4	MW602374	*Yamadazyma dushanensis* CBS 13914	*Y*. *dushanensis*/98.68	17°59′59.2″N 100°15′19.7″E	Site 2: Cho Hae/ Muang	Phrae	1	1.2
FLA2.4	MW602313	*Hannaella luteola* CBS 943	*Ha*. *luteola*/98.99	19°07′16.8″N 98°42′28.9″E	Site 1: Papae/ Mae Taeng	Chiang Mai	1	1.2
FLA26.1	MW602339	*Hannaella pagnoccae* CBS 11142	*Ha*. *pagnoccae*/100.00	19°11′58.0″N 99°31′00.5″E	Site 3: MaeChedi Mai/ Wiang Papao	Chiang Rai	1	1.2
FLA2.1	MW602311	*Hannaella sinensis* CBS 7225	*Ha*. *sinensis*/100.00	19°07′16.8″N 98°42′28.9″E	Site 1: Papae/ Mae Taeng	Chiang Mai	1	1.2
FLA50	MW602380	*Moesziomyces antarcticus* CBS 5955	*Mo*. *antarcticus*/100.00	18°57′46.2″N 99°21′22.3″E	Site 7: Thepsadet/ Doi Saket	Chiang Mai	2	2.4
FLA53	MW602382	*Moesziomyces antarcticus* CBS 5955	*Mo*. *antarcticus*/100.00	18°47′20.4″N 99°15′09.5″E	Site 2: On Nuea/ Mae On	Chiang Mai		
FLA31.5	MW602345	*Papiliotrema flavoscens* CBS 6475	*Pa*. *flavescens*/100.00	19°23′06.9″N 100°41′46.7″E	Site 1: Na Rai Luang/ Song Khwae	Nan	2	2.4
FLA51.1	MW602381	*Papiliotrema flavoscens* CBS 7140	*Pa*. *flavoscens*/100.00	18°57′46.2″N 99°21′22.3″E	Site 8: Thepsadet/ Doi Saket	Chiang Mai		
FLA3.1	MW602314	*Pseudozyma hubeiensis* CBS 10077	*Ps*. *hubeiensis*/99.67	19°07′00.3″N 98°43′35.0″E	Site 2: Papae/ Mae Taeng	Chiang Mai	3	3.5
FLA32.3	MW602350	*Pseudozyma hubeiensis* CBS 10077	*Ps*. *hubeiensis*/100.00	19°14′41.0″N 100°59′52.0″E	Site 1: Sakad/ Pua	Nan		
FLA35.1	MW602353	*Pseudozyma hubeiensis* CBS 10077	*Ps*. *hubeiensis*/99.67	18°07′33.4″N 100°18′57.5″E	Site 1: Suan Khuen/ Muang	Phrae		
FLA36.1	MW602355	*Sporidobolus pararoseus* CBS 491	*Sd*. *pararoseus*/99.30	18°07′02.0″N 100°17′50.9″E	Site 1: Pa Daeng/ Muang	Phrae	1	1.2
FLA31.7	MW602347	*Sporobolomyces bannaensis* CBS 9204	*Sb*. *bannaensis*/100.00	19°23′06.9″N 100°41′46.7″E	Site 1: Na Rai Luang/ Song Khwae	Nan	2	2.4
FLA36.2	MW602356	*Sporobolomyces bannaensis* CBS 9204	*Sb*. *bannaensis*/99.13	18°07′02.0″N 100°17′50.9″E	Site 1: Pa Daeng/ Muang	Phrae		

### 3.2. Phylogenetic results of new yeast species

The sequences obtained in this study were deposited in the GenBank database. Phylogenetic analyses of a combined ITS and D1/D2 dataset of the genera *Metschnikowia* and *Wickerhamiella* were presented in this study. The best substitution models were GTR + I + G for ITS and nrLSU in both analyses. Our phylogenetic analyses revealed that phylograms of the ML and BI analyses in each genus were similar in terms of topology (data not shown). Therefore, the phylogram obtained from the ML analysis was selected and presented in this study.

**Figure 2:**
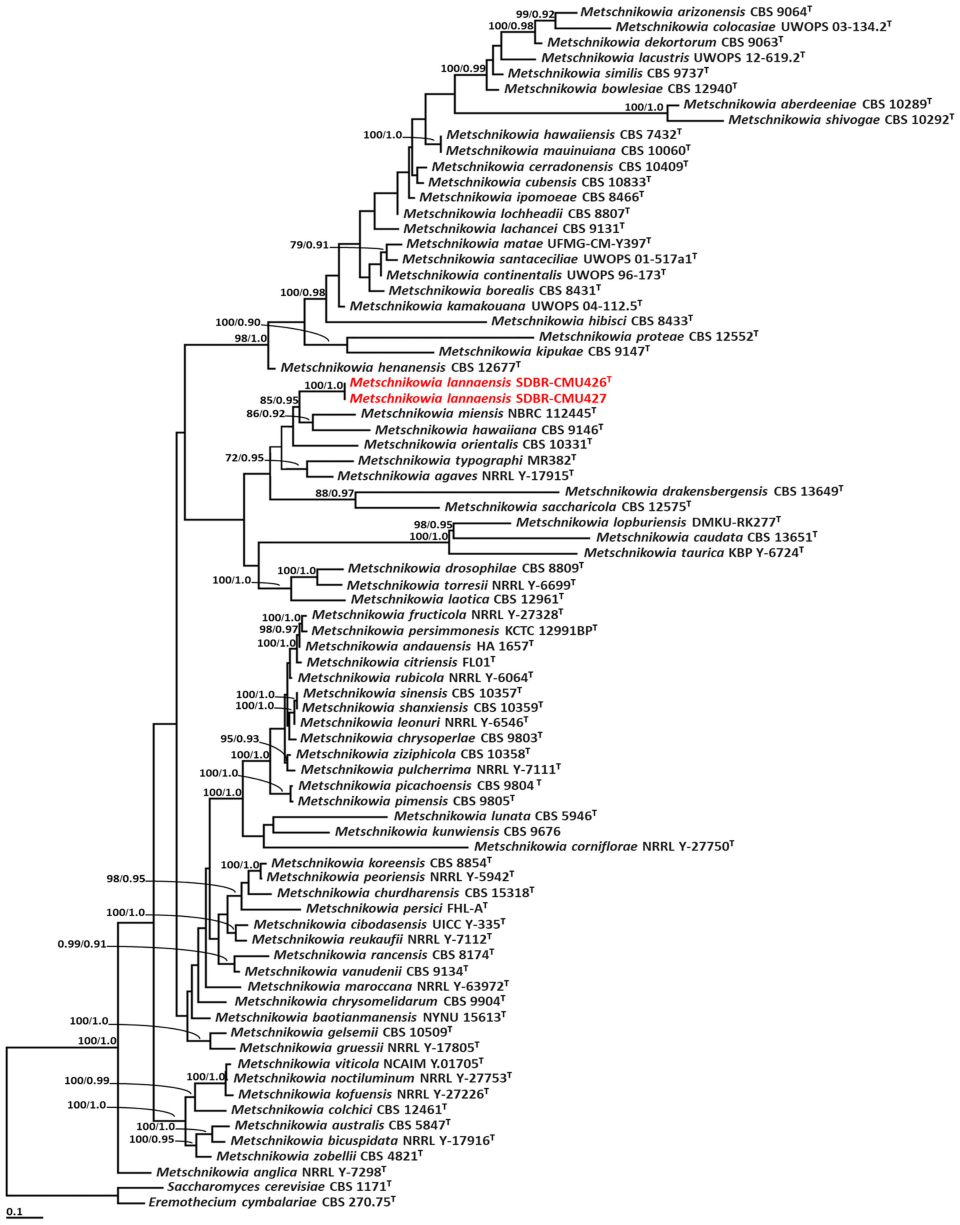
Phylogram derived from maximum likelihood analysis of 77 combined sequences of ITS region and D1/D2 domain of *Metschnikowia* strains. *Eremothecium cymbalariae* CBS 1171 and *Saccharomyces cerevisiae* CBS 270.75 were used as the outgroup. The numbers above branches represent bootstrap percentages (left) and Bayesian posterior probabilities (right). Bootstrap values >70% and Bayesian posterior probabilities >0.90 are shown. The scale bar represents the expected number of nucleotide substitutions per site. Sequences obtained from this study are in red. Type species indicated by superscript “T.”

Phylogenetic analysis of *Metschnikowia* included 77 sequences of *Metschnikowia* and two sequences (*Eremothecium cymbalariae* CBS 1171 and *Saccharomyces cerevisiae* CBS 270.75) of outgroup (Supplementary S3). The aligned dataset was comprised of 1,093 characters including gaps (ITS: 1–664 and D1/D2: 665–1,093). ML analysis of the combined dataset yielded the best scoring tree with a final ML optimization likelihood value of −13,839.8771. A phylogram clearly separated the two *Metschnikowia* strains (SDBR-CMU426 and SDBR-CMU427) (introduced as *M. lannaensis*) obtained in this study from the previously known *Metschnikowia* species with high support values (BS = 100% and PP = 1.0) (Figure 2). *Metschnikowia lannaensis* formed a sister taxon to *M*. *hawaiiana* and *M*. *miensis* with 85% BS and 0.95 PP support values.

**Figure 3:**
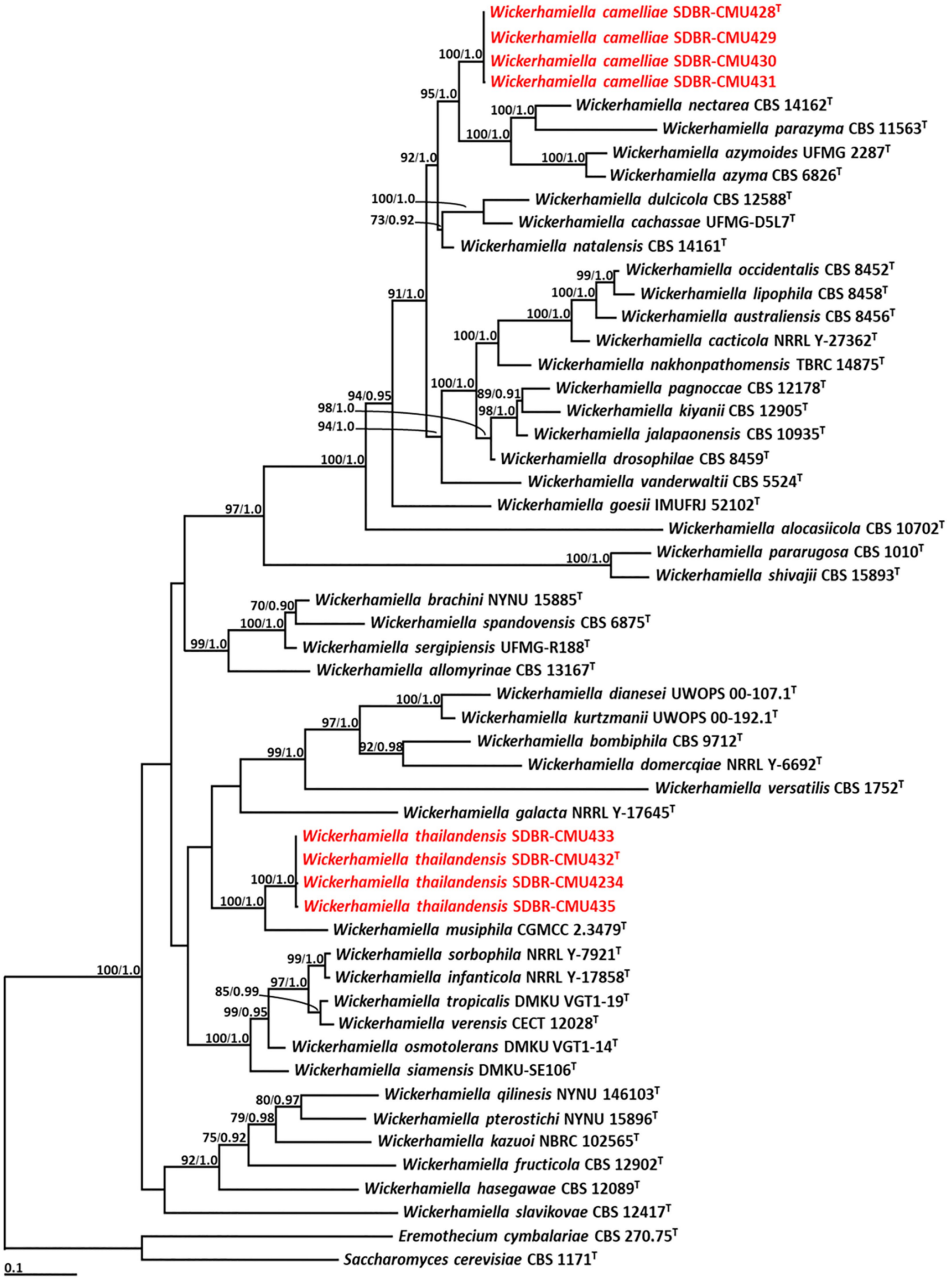
Phylogram derived from maximum likelihood analysis of 52 combined sequences of ITS region and D1/D2 domain of *Wickerhamiella* strains. *Eremothecium cymbalariae* CBS 1171 and *Saccharomyces cerevisiae* CBS 270.75 were used as the outgroup. The numbers above branches represent bootstrap percentages (left) and Bayesian posterior probabilities (right). Bootstrap values >70% and Bayesian posterior probabilities >0.90 are shown. The scale bar represents the expected number of nucleotide substitutions per site. Sequences obtained from this study are in red. Type species indicated by superscript “T.”

Phylogenetic analysis of *Wickerhamiella* used 52 sequences of *Wickerhamiella* and two sequences of the outgroup (*E. cymbalariae* CBS 1171 and *S. cerevisiae* CBS 270.75) (Supplementary S4). The aligned dataset was comprised of 1,249 characters including gaps (ITS: 1–656 and D1/D2: 657–1,249). ML analysis of the combined dataset yielded the best scoring tree with a final ML optimization likelihood value of −14,850.8649. A phylogram clearly separated eight yeast strains into two monophyletic clades with high support values (BS = 100% and PP = 1.0) (Figure 3). The results indicated that four yeast strains, such as SDBR-CMU428, SDBR-CMU429, SDBR-CMU430, and SDBR-CMU431 (introduced as *W. camelliae*), were clearly distinguished from the previously known species of *Wickerhamiella*. It was found that *W. camelliae* formed a sister taxon to *W. azyma*, *W. azymoides*, *W. nectarea*, and *W. parazyma* with 95% BS and 1.0 PP support values. Moreover, four yeast strains in this study, such as SDBR-CMU432, SDBR-CMU433, SDBR-CMU434, and SDBR-CMU435 (described here as *W. thailandensis*) formed a sister clade to *W. musiphila* with high support (BS = 100% and PP = 1.0).

### 3.3. Taxonomic description of novel yeast species

#### 3.3.1. *Metschnikowia lannaensis*

**Figure 4:**
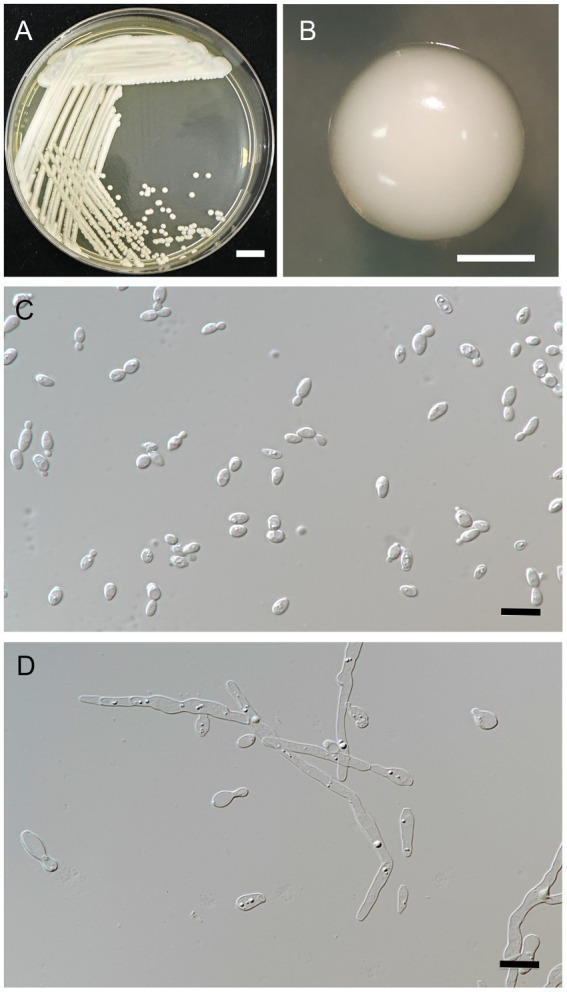
*Metschnikowia lannaensis* FLA8.2^T^ (=SDBR-CMU426^T^, holotype). Culture **(A)**, single colony **(B)**, and cells **(C)** on YMA after incubation at 25°C for 3 days. Pseudohyphae **(D)** on PDA after incubation at 25°C for 4 weeks. Scale bar *A* = 1 cm, *B* = 1 mm, and *C* and *D* = 10 μm.

A. Kanpiengjai, P. Kodchasee, K. Unban, J. Kumla, S. Lumyong, P. Khunnamwong, Sarkar, K. Shetty and C. Khanongnuch sp. nov. (Figure 4).

**MycoBank No.:** MB 845148.

**Etymology:** *lannaensis* (lan.na.en’sis) N.L. fem. adj. lanna, referring to an ancient area of Northern Thailand, where the type strain was isolated.

**Holotype:** FLA8.2^T^ is the holotype of *Metschnikowia lannaensis*. It was isolated from the tea flower of *Camellia sinensis* var. *assamica* collected from the Suthep subdistrict, Muang district, Chiang Mai province, Thailand. It has been preserved in a metabolically inactive state in the culture collection of the Division of Biotechnology, Faculty of Agro-Industry, Chiang Mai University, Chiang Mai, Thailand. The culture ex-type has been permanently deposited in a metabolically inactive state at the Sustainable Development of Biological Resources, Faculty of Science, Chiang Mai University, Chiang Mai, Thailand (SDBR) as SDBR-CMU426.

**Description:** After 3 days at 25°C on YM agar, cells are ovoid (2–3.5 × 5.5–7 μm) and occur singly or in pairs. Budding is multilateral. The colony is a circular form (2–3 mm), white to cream in color, convex, smooth, soft to butyrous, and has an entire margin. Pseudohyphae are formed in slide culture on YMA within 4 weeks at 25°C. Ascospore formation is not observed on PDA, corn meal agar, 5% malt extract agar, Fowell’s acetate agar, YCBY agar, YPD agar, and YM agar at 15°C and 25°C for 4 weeks. Fermentation of glucose is negative. D-Glucose, D-galactose, L-sorbose, sucrose, salicin, raffinose, glycerol (or latent positive), ribitol, D-glucitol, D-mannitol, D-glucono-1,5-lactone, 5-ketogluconic acid, succinate, ethanol, and xylitol (or slow positive) are assimilated, but *N*-acetyl glucosamine, ribose, xylose, L-arabinose, D-arabinose, L-rhamnose, maltose, trehalose, methyl-α-D-glucoside, cellobiose, melibiose, lactose, melizitose, inulin, soluble starch, erythritol, galactitol, *myo*-inositol, 2-ketogluconic acid, D-gluconate, D-glucuronate, D-galacturonic acid, lactate, citrate, and methanol are not assimilated. For the assimilation of nitrogen compounds, growth on ammonium sulfate, ethylamine HCl, and L-lysine is positive, but growth on nitrate, nitrite, and cadaverine is negative.

Growth in the vitamin-free medium is negative. Growth with 0.01% cycloheximide and 0.1% cycloheximide is negative. No growth occurs on media containing 10% NaCl and 16% NaCl. Growth is observed in the presence of 50% glucose, 60% glucose, and at 20, 25, and 30°C, but not at 35 and 37°C. Soluble starch-like extracellular carbohydrates are not produced. The acid formation is negative. Hydrolysis of urea and DBB reaction is negative.

**Additional strains examined:** Thailand, Lampang province, Muang Pan district, Chae Son, isolated from flowers of Assam tea (*C. sinensis* var. *assamica*), January 2016, A. Kanpiengjai, P. Kodchasee, K. Unban, and C. Khanongnuch, SDBR-CMU427.

**GenBank accession number:** holotype FLA8.2^T^ (D1/D2: KY640629, ITS: OM213034); additional strain SDBR-CMU427 (D1/D2: MW542582, ITS: MZ857157).

**Note:** Phylogenetically, *M. lannaensis* formed a sister taxon to *M. hawaiiana* and *M. miensis*. However, *W. lannaensis* could be clearly distinguished from *M. miensis* and *W. hawaiina* by its ability to assimilate D-mannitol in addition to its inability to assimilate *N*-acetyl glucosamine (Shibayama et al., 2020). A pairwise nucleotide comparison of ITS data also indicated that *M. lannaensis* differed from *M. hawaiiana* and *M. miensis* in 19.3% (72/373 bp) and 25.7% (95/369), respectively. In addition, D1/D2 data of *M. lannaensis* differed from *M. hawaiiana* in 11.7% (62/529 bp) and *M. miensis* in 14.5% (78/535 bp).

#### 3.3.2. *Wickerhamiella camelliae*

**Figure 5:**
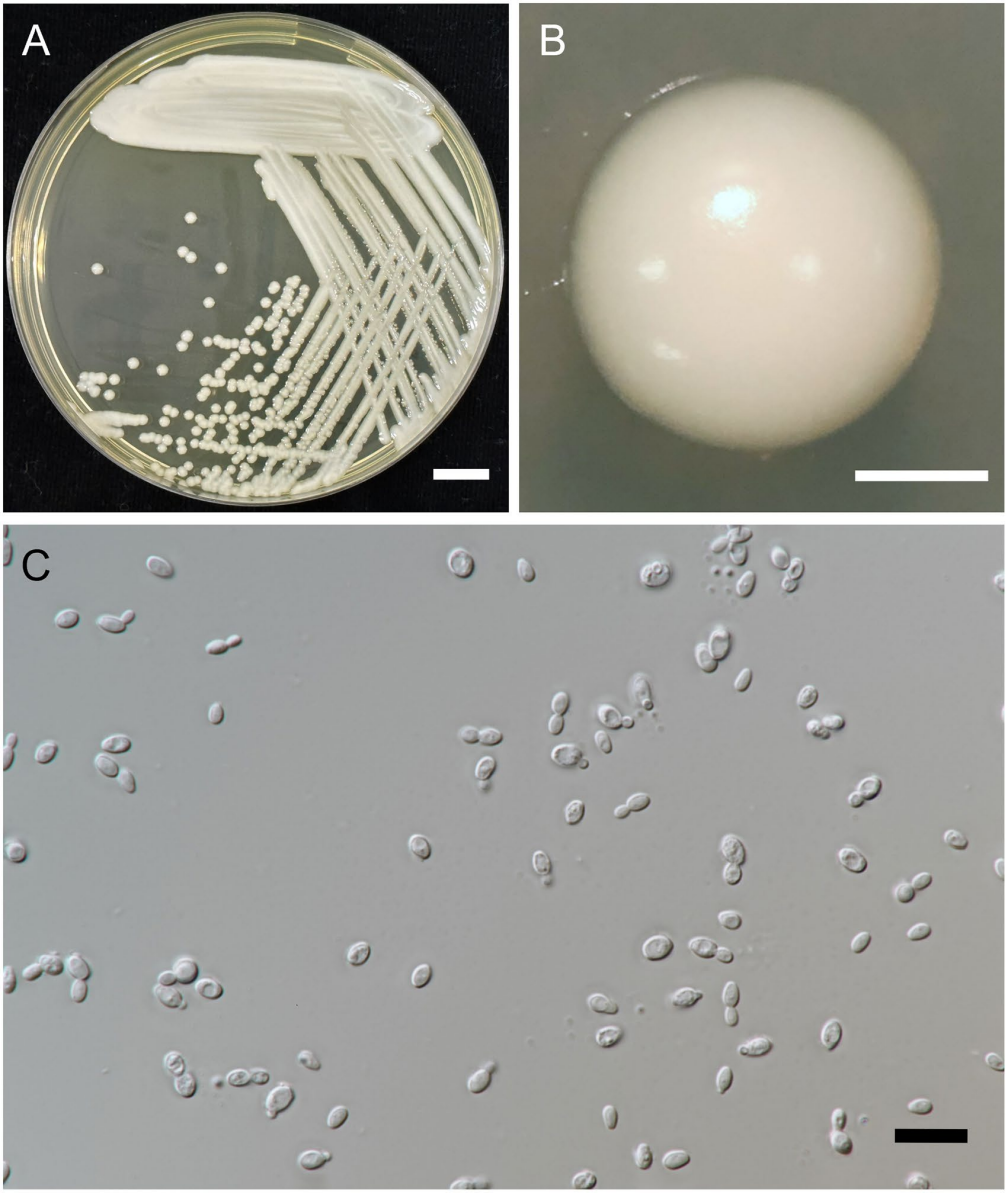
*Wickerhamiella camelliae* FLA5.4^T^ (=SDBR-CMU428^T^, holotype). Culture **(A)**, single colony **(B)**, and cells **(C)** on YMA after incubation at 25°C for 3 days. Scale bar *A* = 1 cm, *B* = 1 mm, and *C* = 10 μm.

A. Kanpiengjai, P. Kodchasee, K. Unban, J. Kumla, S. Lumyong, P. Khunnamwong, D. Sarkar, K. Shetty and C. Khanongnuch sp. nov. (Figure 5).

**MycoBank No.:** MB 845147.

**Etymology:** *camelliae* (*ca.*mel.li’ae) L. fem. adj. camelliae, from the flower of *Camellia sinensis*, referring to the substrate from which the species was isolated.

**Holotype:** FLA5.4^T^ is the holotype of *Wickerhamiella camelliae*. It was isolated from the tea flower of *Camellia sinensis* var. *assamica* collected from the Mae Na subdistrict, Chiang Dao district, Chiang Mai province, Thailand. It has been preserved in a metabolically inactive state in the culture collection of the Division of Biotechnology, Faculty of Agro-Industry, Chiang Mai University, Chiang Mai, Thailand. The culture ex-type has been permanently deposited in a metabolically inactive state at the Sustainable Development of Biological Resources, Faculty of Science, Chiang Mai University, Chiang Mai, Thailand (SDBR) as SDBR-CMU428.

**Description:** After 3 days at 25°C on YM agar, cells are ovoid (2–3 × 5–7 μm) and occur singly or in pairs. Budding is multilateral. The colony is a circular form (2–3 mm), white to cream in color, convex, smooth, and has an entire margin. Pseudohyphae or true hyphae are not formed in slide culture on YMA within 4 weeks at 25°C. Ascospore formation is not observed on PDA, corn meal agar, 5% malt extract agar, Fowell’s acetate agar, YCBY agar, YPD agar, and YM agar at 15°C and 25°C for 4 weeks. Fermentation of glucose is negative. D-Glucose, D-galactose, L-sorbose, xylose, L-arabinose, sucrose, maltose, trehalose, methyl-α-D-glucoside, melizitose, glycerol (or slow positive), D-glucitol, D-mannitol, galactitol, D-glucono-1,5-lactone, 2-ketogluconic acid, succinate, and xylitol are assimilated, but *N*-acetyl glucosamine, D-ribose, L-rhamnose, cellobiose, salicin, melibiose, lactose, raffinose, inulin, soluble starch, erythritol, ribitol, *myo*-inositol, 5-ketogluconic acid, D-glucuronate, D-galacturonic acid, and methanol are not assimilated. Assimilation is variable for DL-lactate, citrate, ethanol, D-arabinose, and D-gluconate. Nitrogen assimilation is positive for ammonium sulfate, ethylamine HCl, cadaverine, and L-lysine but is negative for nitrate and nitrite.

Growth in the vitamin-free medium is negative. Growth with 0.01% cycloheximide and 0.1% cycloheximide is negative. No growth is observed in the presence of 10% NaCl and 16% NaCl. Growth is observed in the presence of 50% glucose, 60% glucose and at 20, 25, and 30°C but not at 35 and 37°C. Soluble starch-like carbohydrates are not produced. The acid formation is negative. Hydrolysis of urea and DBB reaction is negative.

**Additional strains examined:** Thailand, Mae Ai subdistrict, Mae Ai, isolated from the flower of Assam tea (*C. sinensis* var. *assamica*), December 2015, A. Kanpiengjai, P. Kodchasee, K. Unban, and C. Khanongnuch, SDBR-CMU429; Chiang Rai province, Wiang Pa Pao district, Mae Chedi, isolated from the flower of Assam tea (*C. sinensis* var. *assamica*), December 2015, A. Kanpiengjai, P. Kodchasee, K. Unban, and C. Khanongnuch SDBR-CMU430; Phrae province, Muang district, Suan Khuen, isolated from the flower of Assam tea (*C. sinensis* var. *assamica*), March 2016, A. Kanpiengjai, P. Kodchasee, K. Unban, and C. Khanongnuch, SDBR-CMU431.

**GenBank accession number:** holotype FLA5.4^T^ (D1/D2: KY411895, ITS: MZ857161); additional strains SDBR-CMU429 (D1/D2: MW542579, ITS: MZ857162), SDBR-CMU430 (D1/D2: MW542580, ITS: 857163), SDBR-CMU431 (D1/D2: MW542585, ITS: MZ857167).

**Note:** Based on phylogenetic analysis, *W. camelliae* formed a sister taxon to *W. azyma*, *W. azymoides*, *W. nectarea*, and *W. paraazyma*. However, *W. camelliae* could be clearly distinguished from *W. azyma*, *W. azymoides*, *W. nectarea*, and *W. paraazyma* by its inability to assimilate cadaverine. A pairwise nucleotide comparison of ITS data also indicated that *W. camelliae* differed from *W. azyma*, *W. azymoides*, and *W. parazyma* in 17.2% (70/406 bp), 16.2% (66/407 bp), and 19.6% (82/417 bp), respectively. Furthermore, D1/D2 of *W. camelliae* differed from *W. azyma*, *W. azymoides*, *W. nectarea*, and *W. parazyma* in 8.3% (50/556 bp), 9.1% (51/556 bp), 8.1% (45/557 bp), and 12.9% (72/557 bp), respectively.

#### 3.3.3. *Wickerhamiella thailandensis*

**Figure 6:**
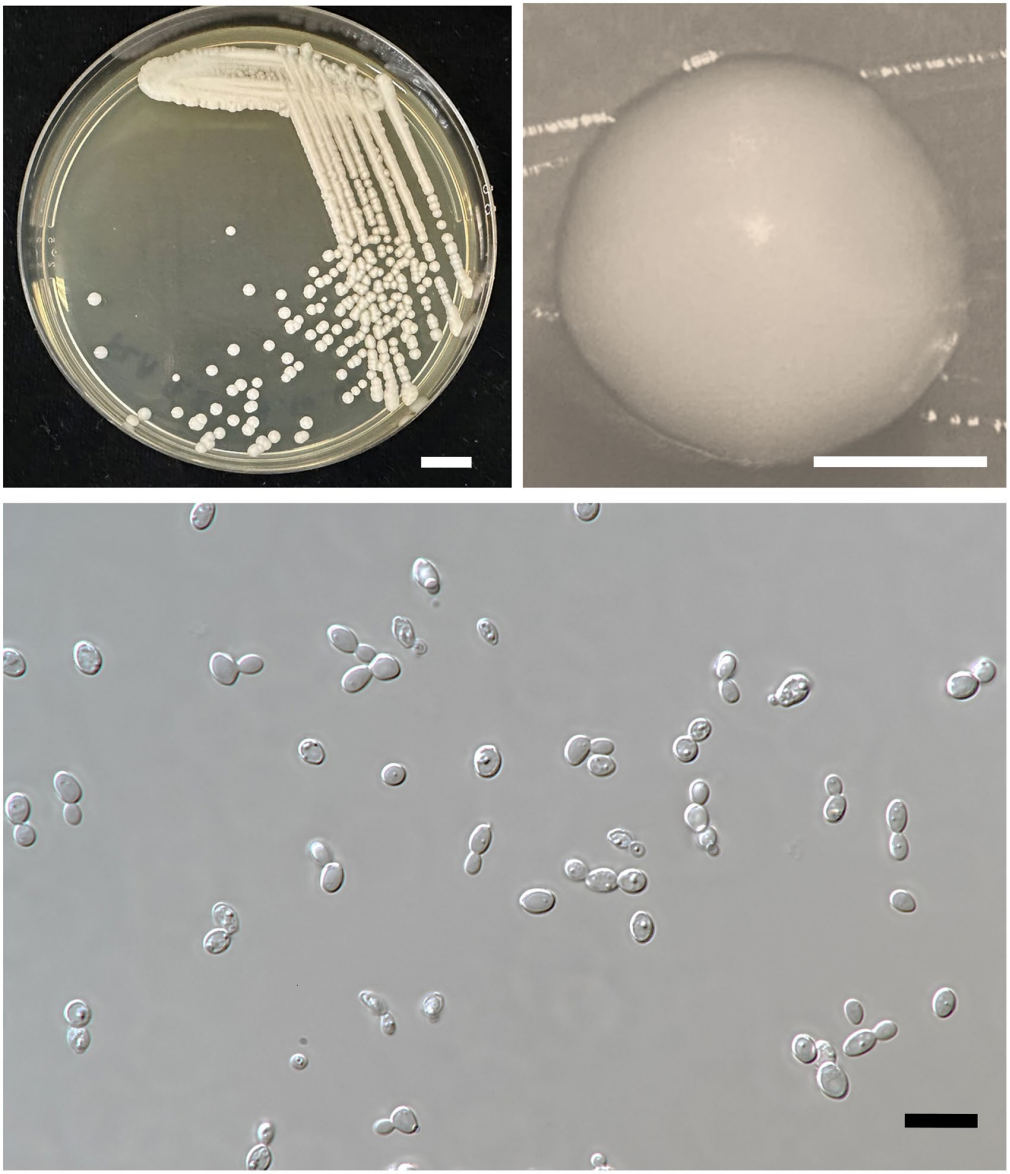
*Wickerhamiella thailandensis* FLA4.2^T^ (=SDBR-CMU432^T^, holotype). Culture **(A)**, single colony **(B)**, and cells **(C)** on YMA after incubation at 25°C for 3 days. Scale bar *A* = 1 cm, *B* = 1 mm, and *C* = 10 μm.

A. Kanpiengjai, P. Kodchasee, K. Unban, J. Kumla, S. Lumyong, P. Khunnamwong, D. Sarkar, K. Shetty and C. Khanongnuch sp. nov. (Figure 6).

**MycoBank No.:** MB 845146.

**Etymology:** *thailandensis* thai.land.en’sis. N.L. fem. adj. thailand, refers to Thailand, where the type strain was isolated.

**Holotype:** FLA4.2^T^ is the holotype of *Wickerhamiella thailandensis*. It was isolated from the tea flower of *Camellia sinensis* var. *assamica* collected from the Pa Pae subdistrict, Mae Taeng district, Chiang Mai province, Thailand. It has been preserved in a metabolically inactive state in the culture collection of the Division of Biotechnology, Faculty of Agro-Industry, Chiang Mai University, Chiang Mai, Thailand. The culture ex-type has been permanently deposited in a metabolically inactive state at the Sustainable Development of Biological Resources, Faculty of Science, Chiang Mai University, Chiang Mai, Thailand (SDBR) as SDBR-CMU432.

**Description:** After 3 days at 25°C on YM agar, cells are subglobose to ovoid (2–3.5 × 5.5–7 μm) and occur singly or in pairs. Budding is multilateral. The colony is a circular form (2–3 mm), white to cream in color, convex, smooth, and has an entire margin. Pseudohyphae or true hyphae are not formed in slide culture on YMA within 4 weeks at 25°C. Ascospore formation is not observed on PDA, corn meal agar, 5% malt extract agar, Fowell’s acetate agar, YCBY agar, YPD agar, and YM agar at 15 and 25°C for 4 weeks. Fermentation of glucose is negative. D-Glucose, D-galactose, L-sorbose, sucrose, raffinose, glycerol (or slow positive), ribitol, D-glucitol, D-mannitol, D-glucono-1,5-lactone, 5-ketogluconic acid (or weak positive), DL-lactate (or slow positive), succinate, ethanol (or weak positive), xylitol (or weak positive), and D-gluconate are assimilated, but *N*-acetyl glucosamine, ribose, xylose, L-arabinose, D-arabinose, L-rhamnose, maltose, trehalose, methyl-α-D-glucoside, cellobiose, salicin, melibiose, lactose, melizitose, inulin, soluble starch, erythritol, galactitol, *myo*-inositol, 2-ketogluconic acid, D-glucuronate, D-galacturonic acid, citrate, and methanol are not assimilated.

Assimilation is variable for D-gluconate. Nitrogen assimilation is positive for ammonium sulfate, ethylamine HCl, lysine, and cadaverine, but is negative for nitrate and nitrite.

Growth in the vitamin-free medium is negative. Growth with 0.01% cycloheximide and 0.1% cycloheximide is negative. Growth in the presence of 50% glucose and 60% glucose is positive, but growth in the presence of 10% NaCl and 16% NaCl is negative. Growth is observed at 20, 25, and 30°C, but not at 35 and 37°C. Soluble starch-like carbohydrates are not produced. The acid formation is negative. Hydrolysis of urea and DBB reaction is negative.

**Additional strains examined:** Thailand, Lampang province, Muang Pan district, Chae Son, isolated from the flower of Assam tea (*C. sinensis* var. *assamica*), January 2016, A. Kanpiengjai, P. Kodchasee, K. Unban, and C. Khanongnuch, SDBR-CMU433, SDBR-CMU434; Chiang Rai province, Thoeng district, Mae Loi, isolated from the flower of Assam tea (*C. sinensis* var. *assamica*), January 2016, A. Kanpiengjai, P. Kodchasee, K. Unban, and C. Khanongnuch, SDBR-CMU435.

**GenBank accession numbers:** holotype FLA4.2^T^ (D1/D2: KY411893, ITS: MZ857160); additional strains SDBR-CMU433 (D1/D2: MW542581, ITS: MZ857164), SDBR-CMU434 (D1/D2: KY640634, ITS: MZ857165), and SDBR-CMU435 (D1/D2: MW542584, ITS: MZ857166).

**Note:** Phylogenetically, *W. thailandensis* formed a sister taxon to *W. musiphila*. However, *W. thailandensis* could be clearly distinguished from *W. musiphila* by its ability to assimilate D-mannitol, D-xylose, and glycerol and its inability to assimilate cadaverine (Wang et al., 2008). Furthermore, ITS and D1/D2 data of *W. thailandensis* differed from *W. musiphila* in ITS 12.3% (49/397 bp) and in D1/D2 5.6% (31/552 bp).

### 3.4. Yeast diversity in tea flowers acquired from the Miang production area of Northern Thailand

**Figure 7:**
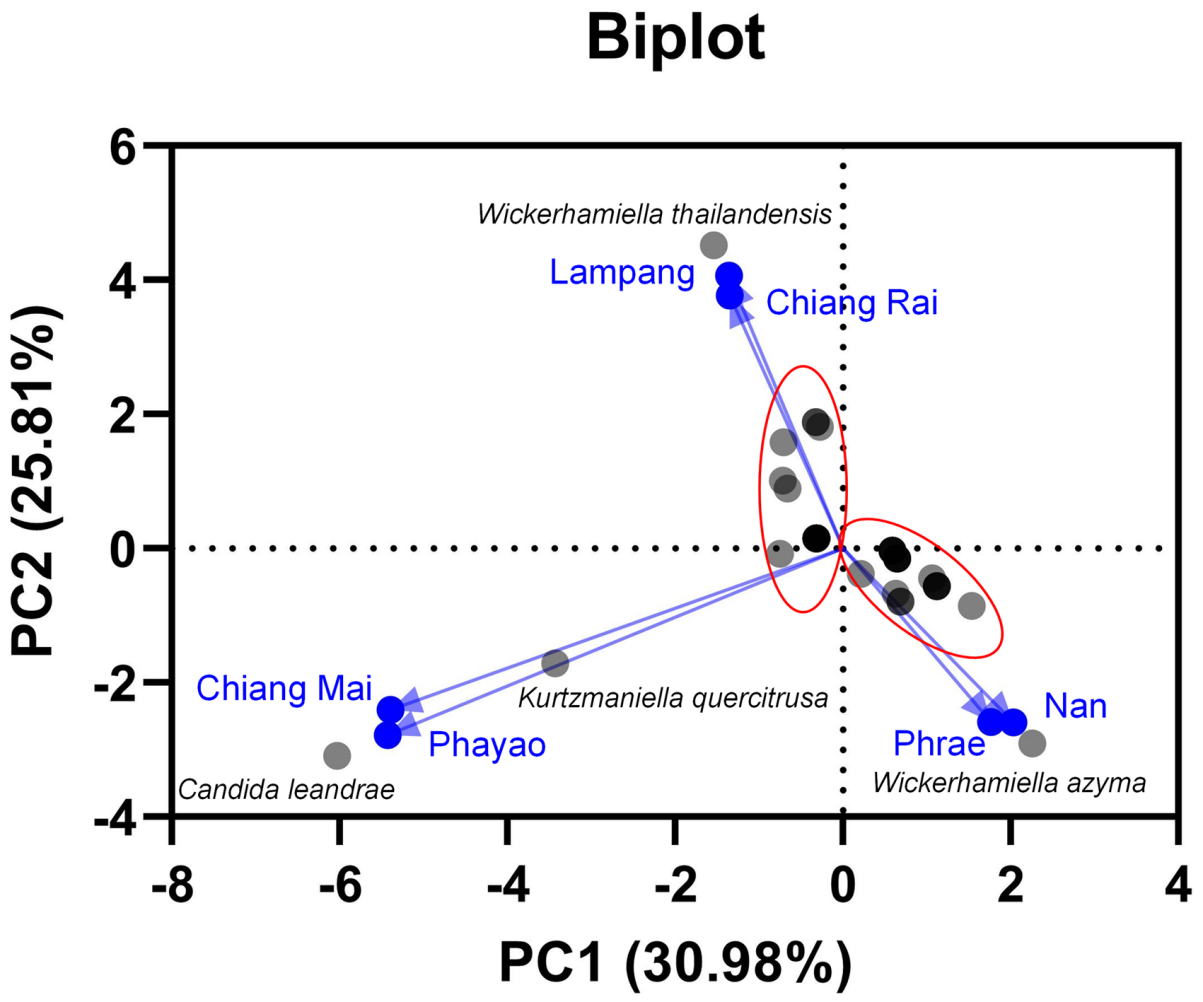
Principal component analyses of yeast species based on the relative abundance of yeast species in tea flower samples, and provinces.

Based on family identification, all 82 yeast strains could be assigned into 12 families. The frequency aligned each species is as follows: Debaryomycetaceae (34.1%), Trichomonascaceae (19.5%), Metschnikowiaceae (17.1%), Ustilaginaceae (6.1%), Pichiaceae (4.9%), Bulleribasdiaceae (3.7%), Phaffomycetaceae (3.7%), Sporidiobolaceae (3.7%), Rhynchogastremataceae (2.4%), Saccharomycetaceae (2.4%), Saccharomycetales (1.2%), and Saccharomycopsidaceae (1.2%). It was observed that in Debaryomycetaceae, 17.1% frequency of occurrence belonged to *Kodamaea*/*Candida* (11.0%), *Kurtzmaniella*/*Candida* (3.7%), and *Candida*/*Lodderomyces* (2.4%) clades. According to the results, *Debaryomyces* spp., *Candida* spp., and *Wickerhamiella* spp. were recorded as the main yeast population found in tea flowers, while *Metschnikowia* spp. was considered the second most abundant yeasts. *Candida leandrae* (10.6%) was the predominant species that was mainly found in samples collected from Chiang Mai. *W. azyma* was the second most abundant species (9.4%) which was acquired from tea flowers collected from Chiang Mai, Phrae, and Nan provinces. *Metschnikowia rancensis*, *W. thailandensis*, and *W. camelliae* were partially found in tea flowers with an equivalent percentage of occurrence (4.7%). The PCA plot reveals three different correlations of yeast species in tea flowers acquired from six provinces of Northern Thailand (Figure 7). The species diversity of yeasts in tea flowers collected from Chiang Mai, Lampang, and Nan provinces showed a high positive correlation with those acquired from Phayao, Chiang Rai, and Phrae, respectively. *Candida leandrae* and *Ku. quercitrusa* were the unique yeast species found in Chiang Mai and Phayao provinces and *W. thailandensis* was mainly found in tea flowers collected from Lampang province. There is a high opportunity to recover *W. azyma* from tea flowers acquired from Phrae and Nan provinces. Other yeast species were generally distributed within tea flowers of six provinces of Northern Thailand. These yeast species could be simply divided into two main groups according to the PCA plot, the first group was yeast species that were widely distributed in tea flowers among Chiang Mai, Phayao, Lampang, and Chiang Rai provinces, the second group was yeast species that were markedly found in tea flowers collected from Phrae and Nan provinces.

### 3.5. Tannin-tolerant yeasts and distribution in Northern Thailand

**Figure 8:**
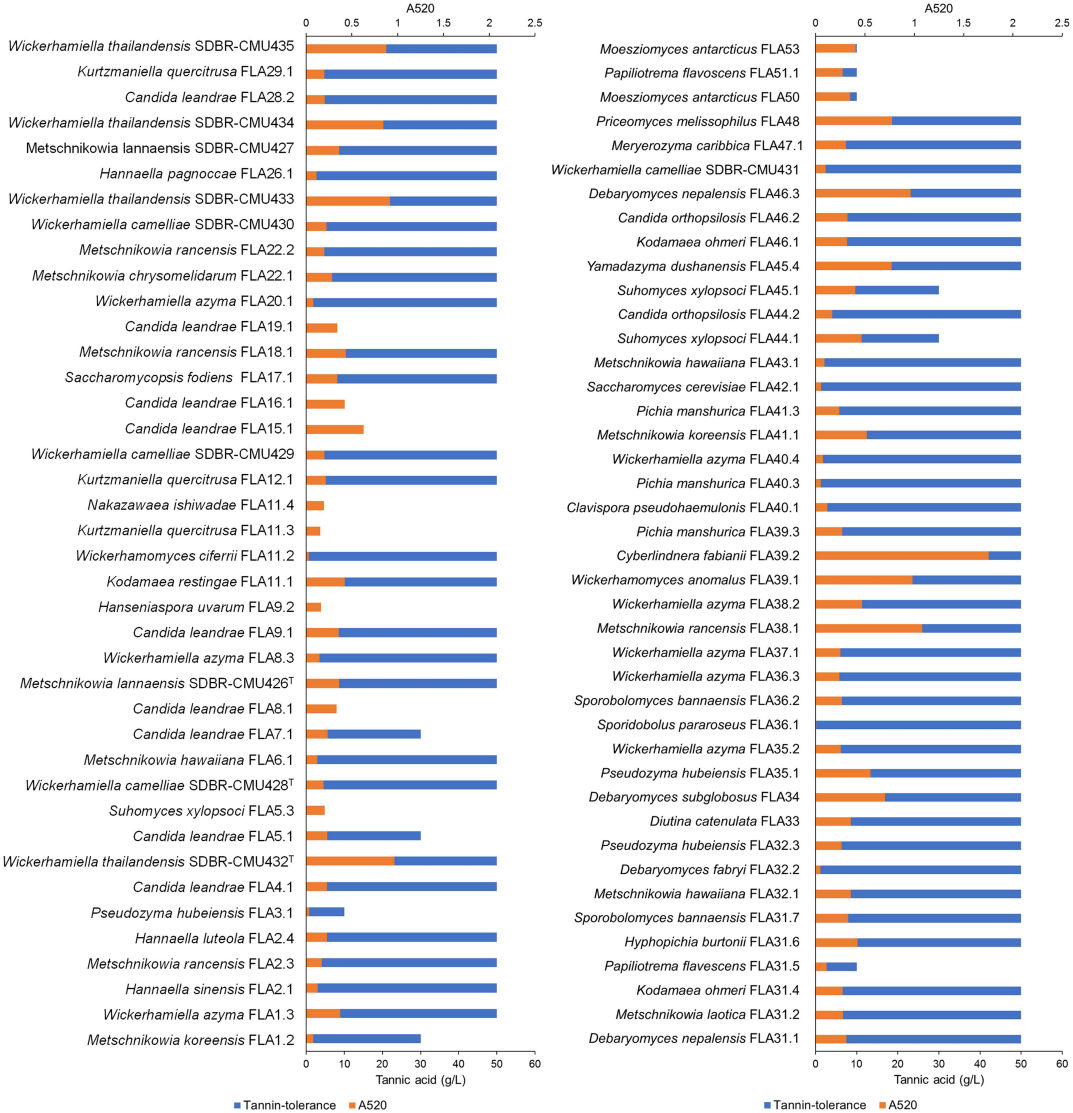
Tannin-tolerant test and tannase activity detection of all yeast strains using the visual reading method. An absorbance value of higher than 0.5, was considered a positive indication of tannase activity.

**Figure 9:**
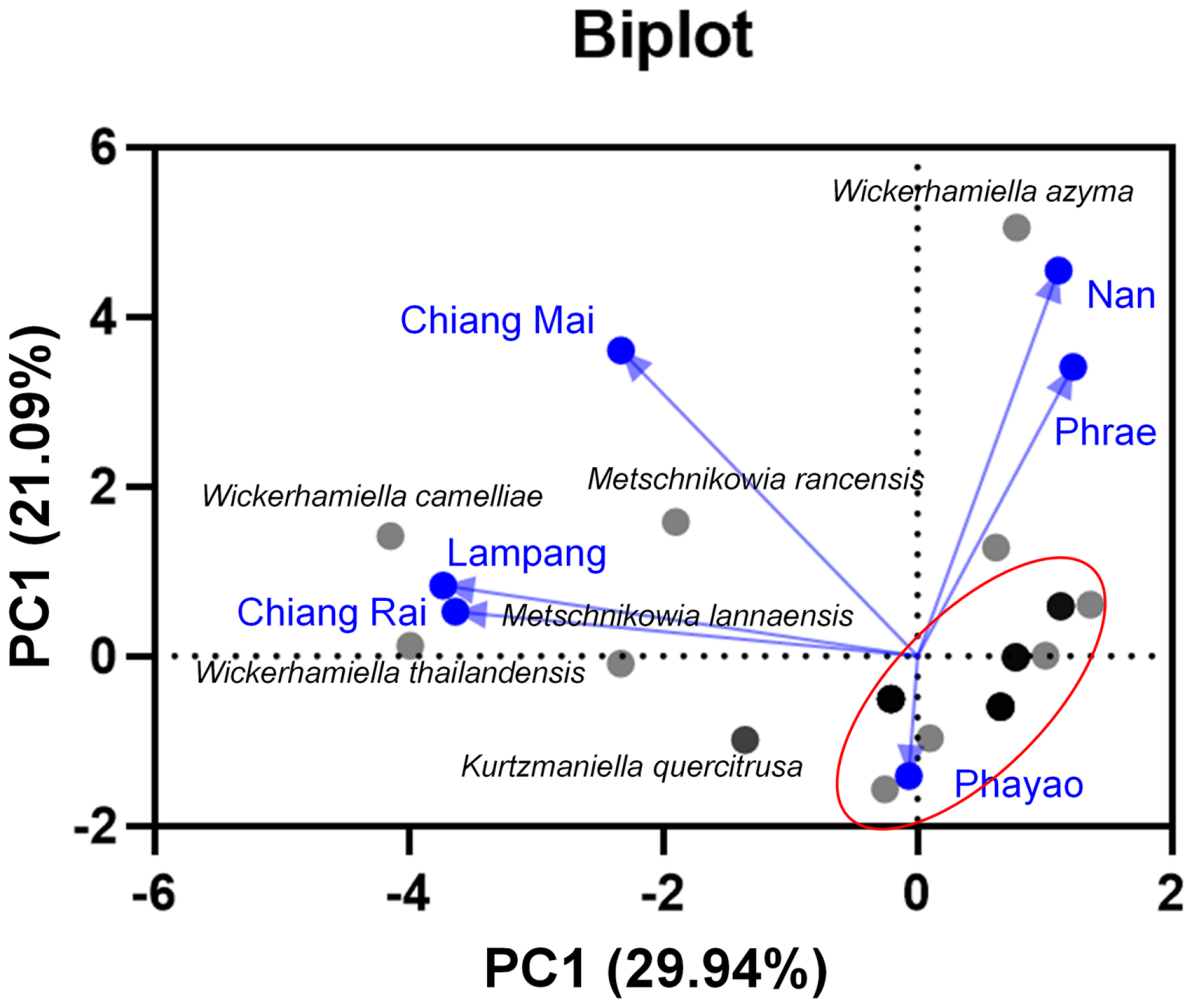
Principal component analyses of tannin-tolerant yeast species based on the relative abundance of yeast species in tea flower samples, and provinces.

All yeast strains were assessed for tannin tolerance using YMA supplemented with different concentrations of tannic acid ranging from 10 to 50 g/L. The results revealed that approximately 78% of the tested yeast strains (64 out of 82 yeast strains) were confirmed for their tannin tolerance at a concentration of 50 g/L tannic acid (Figure 8). These yeasts belong to three main families including Saccharomycetaceae, Metschnikowiaceae, and Trichomonascaceae. A total of 12 out of 64 yeast strains exhibited positive results for tannase activity. These yeasts were *Cy.* (*Cyberlindnera*) *fabianii* FLA39.2, *D. nepalensis* FLA46.3, *D. subglobosus* FLA34, *M. rancensis* FLA38.1, *M. koreensis* FLA41.1, *W. thailandensis* strains FLA4.2., FLA24.1, FLA27.3, and FLA30.2, *Wc.* (*Wickerhamomyces*) *anomalus* FLA39.1, *Pr.* (*Priceomyces*) *melissophilus* FLA48, and *Y. dushanensis* FLA45.4. It was observed that tannin-tolerant ability had species variability. The PCA plot reveals similar correlations of yeast species in tea flowers to those previously described (Figure 9). Interestingly, Phrae and Nan provinces were considered the most diverse areas in upper Northern Thailand for the isolation of tannin-tolerant yeast although the tea plantation area and sampling site were in lower numbers than those in Chiang Mai. *Wickerhamiell azyma* was identified as tannin-tolerant yeast that was uniquely distributed in tea flowers from these areas. The tannin-tolerant yeasts isolated from tea flowers collected from the Phayao province showed poor representation due to the low number of tannin-tolerant yeasts, but the yeast species seem to closely relate with those obtained from samples collected from Phrae and Nan provinces. It was observed that sampling sites of tea flowers from Phayao province align within the eastern part of Northern Thailand where they are in the same location as Phrae and Nan provinces. Here, the distribution of dominant tannin-tolerant yeasts was found within tea flowers collected from Phare, Nan, and Phayao provinces.

## 4. Discussion

The tea plants that are cultivated for Miang production are usually scattered in the forested areas of the mountainous region of Northern Thailand. Generally, the Miang production area is located within the same area of the tea plantation where it belongs to the Miang entrepreneur. Depending on the area of production, two different production processes have been established which include the filamentous fungi growth-based process (FFP) and the non-filamentous fungi growth-based process (NFP; Khanongnuch et al., 2017). In previous studies on the microbial community of Miang fermentation, lactic acid bacteria, *Bacillus* spp., yeasts, and filamentous fungi contributed to physico-chemical changes in Miang directly affected flavor and aroma, health-relevant bioactive compounds, and antioxidant activity of the Miang (Unban et al., 2019; Abdullahi et al., 2021). The evolution of microorganisms associated with Miang fermentation has not yet been elucidated through in-depth studies. Flowers offer favorable microenvironments for yeast growth rather than bacteria (Hausmann et al., 2017) and are recognized as rich sources of novel yeast (De Vega et al., 2017). Unique yeast communities occur to be associated with the rhizosphere, the phylloplane, plant extrudates, necrotic tissues, fruits, and flowers. Like other wild flowers, tea flowers remain a comparatively unexplored reservoir of yeast biodiversity.

The highest number of sampling sites was in Chiang Mai province, followed by Phrae and Nan provinces. However, flowers collected from Phrae and Nan provinces showed more diverse yeast species than those in Chiang Mai. The samples FLA11 and FLA31 were acquired from unexplored locations for Miang production due to difficulty in accessing the tea plantations which are located in high mountains. The incidence of specialist nectar-borne yeast species decreases with increasing pollution index due to the limitation of pollinator foraging behavior; thus, urbanization may restrict the movement of nectar-specialized yeasts (Wehner et al., 2017). Nectar serves as a substrate for the proliferation of yeasts especially Ascomycetes with regard to the various nutrient compositions including lipids, proteins, and amino acids (De Vega et al., 2017). This implication was in accordance with the finding of this study in which most of the yeasts found in tea flowers are specifically ascomycete yeasts. Moreover, three new yeast species, namely, *M. lannaensis*, *W. camelliae*, and *W. thailandensis* isolated from the flower of Assam tea in Northern Thailand were proposed based on identification through molecular phylogenetic and phenotypic (morphological, biochemical, and physiological characteristics) analyses. Based on nucleotide comparisons, all new yeasts described in this study exhibited more than a 1.5% difference in ITS and D1/D2 domain regions with their sister taxa. The yeast strain showing nucleotide substitutions of greater than 1% in the D1/D2 domain of the LSU rRNA gene are usually considered different species (Kurtzman and Robnett, 1997). Therefore, *M. lannaensis*, *W. camelliae*, and *W. thailandensis* can be considered different species. The findings that most yeast strains including three new yeast species were isolated from tea flowers agree with the results from the previous studies which stated that the most frequent yeasts that were recovered from floral nectar and insects belong to the genera *Metschnikowia*, *Starmerella*, *Kodamaea*, and *Wickerhamiella*, but their role on plant–pollinator interactions has not yet been studied (De Vega et al., 2017, 2018). Other yeast genera commonly found in nectar and flora included *Aureobasidium*, *Candida*, *Clavispora*, *Cryptococcus*, *Debaryomyces*, *Hanseniaspora*, *Kodamaea*, *Papiliotrema*, *Rhodotorula*, *Starmerella*, *Sporobolomyces*, and *Wickerhamiella* (Klaps et al., 2020).

The diversity of yeast species in tea flowers was influenced by the sampling site and location. Nan and Phrae provinces were unique areas for the isolation of *W. azyma*, while *C. leandrae* was predominantly found in Chiang Mai. Species diversity of yeasts in flowers is correlated with many factors such as location, the density of flowers (Belisle et al., 2012), pollinators, and other flower-visiting animals (Hausmann et al., 2017; Chappell and Fukami, 2018). Similar to nectar-inhabiting yeast communities between Phrae and Nan provinces may be dependent on geographically distant locations. Therefore, the yeast communities in these provinces were different from those in Chiang Mai.

To evaluate yeasts that may be associated with Miang and the evolution of Miang fermentation, all yeast strains were assessed for tannin-tolerant ability. Based on the results, approximately 78% of total yeast strains were able to tolerate high concentrations at up to 50 g/L tannic acid except for *C. leandrae*, the most abundant yeast found in tea flowers and all strains of *Su.* (*Suhomyces*) *xylosoci*. Some yeasts were identical species reported from both commercial Miang and Miang fermentation processes, i.e., *C. tropicalis*, *Hy. burtonii*, *Me. caribbica*, and *P. manshurica* but the later species including *C. orthopsilosis*, *Cy. fabianii*, *H. uvarum*, *Wc. anomalus* were found only in the Miang fermentation process (Kanpiengjai et al., 2016; Unban et al., 2020a; Kodchasee et al., 2021) and *Wc. anomalus*, a part of the tannin-tolerant yeasts, is capable of producing tannase. Further tannase-producing yeasts found from tea flowers were *Cy. fabianii*, *D. nepalensis*, *D. subglobosus*, *M. rancensis*, *W. thailandensis*, *Pc. melissophilus*, and *Y. dushanensis*. As described in the previous studies, tannin tolerance and tannase-producing ability are good attributes for microorganisms associated with Miang fermentation (Kanpiengjai et al., 2016; Chaikaew et al., 2017; Unban et al., 2020b). The tannin-tolerant yeasts isolated from Miang, *Cy. rhodanensis*, *D. hansenii*, and *S. ruineniae* have been confirmed for their ability to produce cell-associated tannase that played a crucial role in the biotransformation of bioactive phenolic compounds, namely, epigallocatechin gallate (EGCG) and epicatechin gallate (ECG) (Leangnim et al., 2021). For traditional fermented tea leaves such as Pu-erh tea, Fu brick tea, and Laphet-so, it was proposed that properties of yeasts such as *S*. *cerevisiae*, *Blastobotrys adeninivorans*, *Cyberlindnera* sp., and *Candida* sp. may improve the quality of Fu brick tea *via* their enzyme activities toward the degradation of phenolic compounds and producing the sweet substance xylitol and other flavor substances (Li et al., 2017; Zhao et al., 2018). We found coding regions of feruloyl esterase B (FaeB), which is a member of the tannase family, and tannase in whole genome sequences of the reported yeast detected during tea fermentation such as *B*. *adeninivorans* (accession number CAT07374) and *Cy*. *fabianii* (accession number ONH67742). Furthermore, our current research revealed FaeB and tannase in the transcriptome of tannin-tolerant and tannase-producing yeasts isolated from Miang, namely, *Cy. rhodanensis* and *Sd. ruineniae* (data not shown). Considering the catalytic region of all above-mentioned tannases and FaeBs, GCSXGG sequence at the catalytic region and CS-D-HC motif as the catalytic triads of the enzymes are always conserved for fungal and yeast tannases that have high specificity toward catechins and condensed tannins from the plant cell wall (Suzuki et al., 2014; Dong et al., 2021). It is believed that the coding regions of these enzymes may either be conserved within species or varied among different yeast genera. Although the main yeasts in Miang and Miang fermentation processes were found to be *C. ethanolica* and *P. manshurica*, it is assumed that a part of tannin-tolerant yeasts isolated from tea flowers which are conserved within Miang fermentation may be alternatively associated with the fermentation by playing roles in the production of Miang fermentation such as promoting an appropriate environment for tannase-producing yeasts and other microorganisms associated with developing steamed tea leaves into Miang, and enhancement of flavor and aroma to serve a unique Miang product of each Miang production area.

In summary, we present the yeast ecology of tea flowers and suggest that it is partially associated with Miang fermentation based on their tannin tolerance and tannase-producing ability. The yeast ecology is mainly restricted to the flower habitat, and this strengthens the relevance of plant–pollinator interactions. It can be further concluded that tea flowers are a potential source of tannin-tolerant and tannase-producing yeasts, which will shed more light as the important microbial source for the development of Miang fermentation and the production of bioactive compounds from Miang.

## Data availability statement

The datasets presented in this study can be found in online repositories. The names of the repository/repositories and accession number(s) can be found in the article/Supplementary material.

## Author contributions

AK, CK, JK, and SL were responsible for conceptualization and funding acquisition. AK, CK, KU, and PKo collected the species, performed the morphological analysis, and molecular phylogenetic analyses. PKh, AK, and PKo performed biochemical and physiological analyses. AK wrote the first draft. AK, CK, KU, JK, and PKh wrote, reviewed, and edited the manuscript. DS and KS gave some suggestions for English improvement and the flow of concepts. All authors contributed to the article and approved the submitted version.

## Funding

This study was supported financially by the National Research Council of Thailand (NRCT) and also funding from the Royal Golden Jubilee Ph.D. (RGJ) Programme (grant no. PHD/0137/2560). We are also grateful to Chiang Mai University for financial support *via* a postdoctoral fellowship.

## Conflict of interest

The authors declare that the research was conducted in the absence of any commercial or financial relationships that could be construed as a potential conflict of interest.

## Publisher’s note

All claims expressed in this article are solely those of the authors and do not necessarily represent those of their affiliated organizations, or those of the publisher, the editors and the reviewers. Any product that may be evaluated in this article, or claim that may be made by its manufacturer, is not guaranteed or endorsed by the publisher.

## Footnotes

^1^ http://blast.ncbi.nlm.nih.gov
