# Supplementary material for: Three new yeast species from flowers of Camellia sinensis var. assamica collected in Northern Thailand and their tannin tolerance characterization
Source: Front Microbiol. 2023 Feb 16;14:1043430. doi: 10.3389/fmicb.2023.1043430 (PMC9978478; doi:10.3389/fmicb.2023.1043430)
Supplement: Supplementary file 1 [file Data_Sheet_1.docx]

**Supplementary Table S1** Location from which the samples were retrieved and sampling site, and quantity of yeast isolates in each sample.

| **Date achieved** | **Code** | **Location** | **Sites (subdistrict/district)** | **Province** | **Isolates/sample** |
| --- | --- | --- | --- | --- | --- |
| 11 Dec 2015 | FLA1 | 18°47'08.5"N 100°39'37.5"E | Site 1: Ruang/ Muang | Nan | 2 |
|  | FLA2 | 19°07'16.8"N 98°42'28.9"E | Site 1: Papae/ Mae Taeng | Chiang Mai | 3 |
|  | FLA3 | 19°07'00.3"N 98°43'35.0"E | Site 2: Papae/ Mae Taeng | Chiang Mai | 1 |
|  | FLA4 | 19°07'00.3"N 98°43'35.0"E | Site 3: Papae/ Mae Taeng | Chiang Mai | 2 |
|  | FLA5 | 19°44'01.9"N 98°54'04.1"E | Site 1: Mae Na/ Chiang Dao | Chiang Mai | 3 |
|  | FLA6 | 18°50'29.3"N 98°54'15.9"E | Site 1: Suthep/ Muang | Chiang Mai | 1 |
|  | FLA7 | 18°50'29.3"N 98°54'15.9"E | Site 2: Suthep/ Muang | Chiang Mai | 1 |
|  | FLA8 | 18°50'29.3"N 98°54'15.9"E | Site 3: Suthep/ Muang | Chiang Mai | 3 |
|  | FLA9 | 18°50'29.3"N 98°54'15.9"E | Site 4: Suthep/ Muang | Chiang Mai | 2 |
| 20 Dec 2015 | FLA10 | 18°50'29.3"N 98°54'15.9"E | Site 5: Suthep/ Muang | Chiang Mai | 0 |
| 23 Dec 2015 | FLA11 | 19°05'43.3"N 99°10'54.5"E | Site 1: Longkhod/ Phrao | Chiang Mai | 4 |
|  | FLA12 | 19°05'43.3"N 99°10'54.5"E | Site 1: Mae Ai/ Mai Ai | Chiang Mai | 1 |
|  | FLA13 | 20°05'09.9"N 99°17'11.0"E | Site 2: Mae Ai/ Mae Ai | Chiang Mai | 1 |
|  | FLA14 | 20°05'09.9"N 99°17'11.0"E | Site 3: Mae Ai/ Mai Ai | Chiang Mai | 0 |
| 27 Dec 2015 | FLA15 | 18°57'41.2"N 99°14'06.9"E | Site 1: Pa Miang/ Doi Saket | Chiang Mai | 1 |
|  | FLA16 | 18°57'46.2"N 99°21'22.3"E | Site 1: Thepsadet/ Doi Saket | Chiang Mai | 1 |
|  | FLA17 | 18°55'15.8"N 99°19'49.0"E | Site 2: Thepsadet/ Doi Saket | Chiang Mai | 1 |
|  | FLA18 | 18°57'42.3"N 99°14'04.9"E | Site 2: Pa Miang/ Doi Saket | Chiang Mai | 1 |
|  | FLA19 | 18°57'46.2"N 99°21'22.3"E | Site 3: Thepsadet/ Doi Saket | Chiang Mai | 1 |
|  | FLA20 | 18°55'18.7"N 99°19'54.4"E | Site 4: Thepsadet/ Doi Saket | Chiang Mai | 1 |
|  | FLA21 | 18°59'34.7"N 99°20'35.7"E | Site 5: Thepsadet/ Doi Saket | Chiang Mai | 0 |
| 30 Dec 2015 | FLA22 | 19°05'16.8"N 99°22'29.6"E | Site 1: Mae Chedi Mai/ Wiang Papao | Chiang Rai | 2 |
|  | FLA23 | 19°05'16.8"N 99°22'29.6"E | Site 2: Mae Chedi Mai/ Wiang Papao | Chiang Rai | 0 |
| 7 Jan 2016 | FLA24 | 18°49'43.4"N 99°23'14.6"E | Site 1: Chae Son/ Muang Pan | Lampang | 1 |
|  | FLA25 | 18°57'41.2"N 99°14'06.9"E | Site 3: Pa Miang/ Doi Saket | Chiang Mai | 0 |
|  | FLA26 | 19°11'58.0"N 99°31'00.5"E | Site 3: Mae Chedi Mai/ Wiang Papao | Chiang Rai | 1 |
|  | FLA27 | 18°49'43.4"N 99°23'14.6"E | Site 2: Chae Son/ Muang Pan | Lampang | 2 |

**Supplementary Table S1** (continued)

| **Date achieved** | **Code** | **Location** | **Sites (subdistrict/district)** | **Province** | **Isolate/sample** |
| --- | --- | --- | --- | --- | --- |
| 15 Jan 2016 | FLA28 | 19°35'19.1"N 100°22'30.0"E | Site 1: Phu Sang/ Chiang Kham | Phayao | 2 |
|  | FLA29 | 19°14'18.4"N 100°19'50.1"E | Site 1: Oi/ Pong | Phayao | 1 |
|  | FLA30 | 19°34'00.7"N 100°02'35.8"E | Site 1: Mae Loi/ Thoeng | Chiang Rai | 1 |
|  | FLA31 | 19°23'06.9"N 100°41'46.7"E | Site 1: Na Rai Luang/ Song Khwae | Nan | 6 |
| 21 Feb 2016 | FLA32 | 19°14'41.0"N 100°59'52.0"E | Site 1: Sakad/ Pua | Nan | 3 |
|  | FLA33 | 19°14'41.0"N 100°59'52.0"E | Site 2: Sakad/ Pua | Nan | 1 |
|  | FLA34 | 19°14'41.0"N 100°59'52.0"E | Site 3: Sakad/ Pua | Nan | 1 |
|  | FLA35 | 18°07'33.4"N 100°18'57.5"E | Site 1: Suan Khuen/ Muang | Phrae | 2 |
|  | FLA36 | 18°07'02.0"N 100°17'50.9"E | Site 1: Pa Daeng/ Muang | Phrae | 3 |
| 15 Mar 2016 | FLA37 | 18°47'08.5"N 100°39'37.5"E | Site 2: Ruang/ Muang | Nan | 1 |
|  | FLA38 | 18°47'08.5"N 100°39'37.5"E | Site 3: Ruang/ Muang | Nan | 2 |
|  | FLA39 | 18°07'02.0"N 100°17'50.9"E | Site 2: Pa Daeng/ Muang | Phrae | 3 |
|  | FLA40 | 18°07'02.0"N 100°17'50.9"E | Site 3: Pa Daeng/ Muang | Phrae | 3 |
|  | FLA41 | 18°07'02.0"N 100°17'50.9"E | Site 4: Pa Daeng/ Muang | Phrae | 2 |
|  | FLA42 | 18°07'02.0"N 100°17'50.9"E | Site 5: Pa Daeng/ Muang | Phrae | 1 |
|  | FLA43 | 18°07'02.0"N 100°17'50.9"E | Site 6: Pa Daeng/ Muang | Phrae | 1 |
|  | FLA44 | 17°59'59.2"N 100°15'19.7"E | Site 1: Cho Hae/ Muang | Phrae | 2 |
|  | FLA45 | 17°59'59.2"N 100°15'19.7"E | Site 2: Cho Hae/ Muang | Phrae | 2 |
|  | FLA46 | 18°07'33.4"N 100°18'57.5"E | Site 2: Suan Khuen/ Muang | Phrae | 4 |
| 5 May 2016 | FLA47 | 18°55'24.7"N 99°12'03.5"E | Site 4: Pa Miang/ Doi Saket | Chiang Mai | 1 |
|  | FLA48 | 18°55'24.7"N 99°12'03.5"E | Site 5: Pa Miang/ Doi Saket | Chiang Mai | 1 |
|  | FLA49 | 18°57'46.2"N 99°21'22.3"E | Site 6: Thepsadet/ Doi Saket | Chiang Mai | 0 |
|  | FLA50 | 18°57'46.2"N 99°21'22.3"E | Site 7: Thepsadet/ Doi Saket | Chiang Mai | 1 |
|  | FLA51 | 18°57'46.2"N 99°21'22.3"E | Site 8: Thepsadet/ Doi Saket | Chiang Mai | 1 |
|  | FLA52 | 18°47'20.4"N 99°15'09.5"E | Site 1: On Nuea/ Mae On | Chiang Mai | 0 |
|  | FLA53 | 18°47'20.4"N 99°15'09.5"E | Site 2: On Nuea/ Mae On | Chiang Mai | 1 |

**Supplementary Table S2** Identification of yeast strains isolated from Miang tea flower and their GenBank accession numbers of the D1/D2 domain of LSU rRNA gene.

| **No.** | **Isolate** | **D1/D2 sequence (nt)** | **Similarity** | **CBS type strain** | **CBS type strain Accession no.** | **Identification results** | **Accession no.** | **No. of sample found for each species** | **% Frequency of occurrence** | | |
| --- | --- | --- | --- | --- | --- | --- | --- | --- | --- | --- | --- |
|  | **Phylum Ascomycota** | | | | | | | | |  |  |
| 1 | FLA4.1 | 492 | 99.8 | *Candida leandrae* CBS 9735 | KY106547 | *Candida leandrae* | MW602315 | 9 | 10.6 | | |
| 2 | FLA5.1 | 492 | 99.8 | *Candida leandrae* CBS 9735 | KY106547 | *Candida leandrae* | MW602316 |  |  | | |
| 3 | FLA7.1 | 492 | 99.8 | *Candida leandrae* CBS 9735 | KY106547 | *Candida leandrae* | MW602319 |  |  | | |
| 4 | FLA8.1 | 492 | 99.8 | *Candida leandrae* CBS 9735 | KY106547 | *Candida leandrae* | MW602320 |  |  | | |
| 5 | FLA9.1 | 492 | 99.8 | *Candida leandrae* CBS 9735 | KY106547 | *Candida leandrae* | MW602322 |  |  | | |
| 6 | FLA15.1 | 492 | 99.8 | *Candida leandrae* CBS 9735 | KY106547 | *Candida leandrae* | MW602331 |  |  | | |
| 7 | FLA16.1 | 492 | 99.8 | *Candida leandrae* CBS 9735 | KY106547 | *Candida leandrae* | MW602332 |  |  | | |
| 8 | FLA19.1 | 492 | 99.8 | *Candida leandrae* CBS 9735 | KY106547 | *Candida leandrae* | MW602335 |  |  | | |
| 9 | FLA28.2 | 492 | 99.8 | *Candida leandrae* CBS 9735 | KY106547 | *Candida leandrae* | MW602340 |  |  | | |
| 10 | FLA44.2 | 571 | 100 | *Candida orthopsilosis* CBS 10744 | MK394126 | *Candida orthopsilosis* | MW602372 | 2 | 2.4 | | |
| 11 | FLA46.2 | 570 | 100 | *Candida orthopsilosis* CBS 10744 | MK394126 | *Candida orthopsilosis* | MW602376 |  |  | | |
| 12 | FLA40.1 | 505 | 99.21 | *Clavispora pseudohaemulonis* CBS 10004 | MK394152 | *Clavispora pseudohaemulonis* | MW602364 | 1 | 1.2 | | |
| 13 | FLA39.2 | 570 | 100 | *Cyberlindnera fabianii* CBS 5640 | MK394133 | *Cyberlindnera fabianii* | MW602345 | 1 | 1.2 | | |
| 14 | FLA32.2 | 570 | 100 | *Debaryomyces fabryi* CBS 789 | U94927 | *Debaryomyces fabryi* | MW602349 | 1 | 1.2 | | |
| 15 | FLA31.1 | 568 | 100 | *Debaryomyces nepalensis* CBS 7761 | KY107576 | *Debaryomyces natalensis* | MW602342 | 2 | 2.4 | | |
| 16 | FLA46.3 | 570 | 100 | *Debaryomyces nepalensis* CBS 7761 | KY107576 | *Debaryomyces nepalensis* | MW602377 |  |  | | |
| 17 | FLA34 | 570 | 100 | *Debaryomyces subglobosus* CBS 792 | EU816211 | *Debaryomyces subglobosus* | MW602352 | 1 | 1.2 | | |
| 18 | FLA33 | 465 | 100 | *Diutina catenulata* CBS 565 | MK394156 | *Diutina catenulata* | MW602351 | 1 | 1.2 | | |
| 19 | FLA9.2 | 572 | 99.65 | *Hanseniaspora uvarum* CBS 104 | KY107838 | *Hanseniaspora uvarum* | MW602323 | 1 | 1.2 | | |
| 20 | FLA31.6 | 524 | 100 | *Hyphopichia buttonii* CBS 4571 | GQ389658 | *Hyphopichia burtonii* | MW602346 | 1 | 1.2 | | |
| 21 | FLA31.4 | 493 | 100 | *Kodamaea ohmeri* CBS 5367 | MK394144 | *Kodamaea ohmeri* | MW602344 | 2 | 2.4 | | |
| 22 | FLA46.1 | 493 | 100 | *Kodamaea ohmeri* CBS 5367 | MK394144 | *Kodamaea ohmeri* | MW602375 |  |  | | |
| 23 | FLA11.1 | 493 | 98.58 | *Kodamaea restingae* CBS 8492 | AF059667 | *Kodamaea restingae* | MW602325 | 1 | 1.2 | | |
| 24 | FLA11.3 | 565 | 100 | *Kurtzmaniella quercitrusa* CBS 4412 | MK394107 | *Kurtzmaniella quecitrusa* | MW602327 | 3 | 3.5 | | |
| 25 | FLA12.1 | 565 | 99.82 | *Kurtzmaniella quercitrusa* CBS 4412 | MK394107 | *Kurtzmaniella quecitrusa* | MW602330 |  |  | | |
| 26 | FLA29.1 | 565 | 100 | *Kurtzmaniella quercitrusa* CBS 4412 | MK394107 | *Kurtzmaniella quecitrusa* | MW602341 |  |  | | |
| 27 | FLA47.1 | 570 | 100 | *Meryerozyma caribbica* CBS 9966 | MH54591 | *Meryerozyma caribbica* | MW602378 | 1 | 1.2 | | |
| **Supplementary Table S2** (Continued) | | | | | | | | | | |  |
| **No.** | **Isolate** | **D1/D2 sequence (nt)** | **Similarity** | **CBS type strain** | **CBS type strain Accession no.** | **Identification results** | **Accession no.** | **No. of sample found for each species** | **% Frequency of occurrence** | | |
| 28 | FLA22.1 | 479 | 99.58 | *Metschnikowia chrysomelidarum* CBS 9904 | KY106396 | *Metschnikowia chrysomelidarum* | MW602337 | 1 | 1.2 | | |
| 29 | FLA6.1 | 523 | 99.8 | *Metschnikowia hawaiiana* CBS 9146 | NG060810 | *Metschnikowia hawaiiana* | MW602318 | 3 | 3.5 | | |
| 30 | FLA32.1 | 523 | 100 | *Metschnikowia hawaiiana* CBS 9146 | NG060810 | *Metschnikowia hawaiiana* | MW602348 |  |  | | |
| 31 | FLA43.1 | 523 | 100 | *Metschnikowia hawaiiana* CBS 9146 | NG060810 | *Metschnikowia hawaiiana* | MW602370 |  |  | | |
| 32 | FLA1.2 | 512 | 100 | *Metschnikowia koreensis* CBS 8854 | NG058340 | *Metschnikowia koreensis* | MW602309 | 2 | 2.4 | | |
| 33 | FLA41.1 | 512 | 100 | *Metschnikowia koreensis* CBS 8854 | NG058340 | *Metschnikowia koreensis* | MW602367 |  |  | | |
| 34 | FLA31.2 | 529 | 99.2 | *Metschnikowia laotica*  CBS 12961 | JX515978 | *Metschnikowia laotica* | MW602343 | 1 | 1.2 | | |
| 35 | FLA2.3 | 509 | 100 | *Metschnikowia rancensis* CBS 8174 | NG055309 | *Metschnikowia rancensis* | MW602312 | 4 | 4.7 | | |
| 36 | FLA18.1 | 509 | 99.8 | *Metschnikowia rancensis* CBS 8174 | NG055309 | *Metschnikowia rancensis* | MW602334 |  |  | | |
| 37 | FLA22.2 | 509 | 99.8 | *Metschnikowia rancensis* CBS 8174 | NG055309 | *Metschnikowia rancensis* | MW602338 |  |  | | |
| 38 | FLA38.1 | 509 | 99.8 | *Metschnikowia rancensis* CBS 8174 | NG055309 | *Metschnikowia rancensis* | MW602359 |  |  | | |
| 39 | FLA11.4 | 570 | 100 | *Nakazawaea ishiwadae* CBS 6022 | NG055159 | *Nakazawaea ishiwadae* | MW602328 | 1 | 1.2 | | |
| 40 | FLA8.2 | 526 | 89.35 | *Metschinikowia hawiiana* CBS 9146 | NG060810 | **SDBR-CMU426** | KY640629 | 2 | 2.4 | | |
| 41 | FLA27.1 | 526 | 89.16 | *Metschinikowia hawiiana* CBS 9146 | NG060810 | **SDBR-CMU427** | MW542582 |  |  | | |
| 42 | FLA5.4 | 557 | 96.95 | *Wickerhamiella natalensis* CBS 14161 | KT158538 | **SDBR-CMU428** | KY411895 | 4 | 4.7 | | |
| 43 | FLA13.1 | 526 | 96.59 | *Wickerhamiella natalensis* CBS 14161 | KT158538 | **SDBR-CMU429** | MW542579 |  |  | | |
| 44 | FLA23.2 | 557 | 96.95 | *Wickerhamiella natalensis* CBS 14161 | KT158538 | **SDBR-CMU430** | MW542580 |  |  | | |
| 45 | FLA46.4 | 557 | 96.95 | *Wickerhamiella natalensis* CBS 14161 | KT158538 | **SDBR-CMU431** | MW542585 |  |  | | |
| 46 | FLA4.2 | 552 | 94.38 | *Wickerhamiella musiphila* CBS10697 | NG055366 | **SDBR-CMU432** | KY411893 | 4 | 4.7 | | |
| 46 | FLA24.1 | 552 | 94.38 | *Wickerhamiella musiphila* CBS 10697 | NG055366 | **SDBR-CMU433** | MW542581 |  |  | | |
| 47 | FLA27.3 | 552 | 94.57 | *Wickerhamiella musiphila* CBS 10697 | NG055366 | **SDBR-CMU434** | KY640634 |  |  | | |
| 48 | FLA30.2 | 552 | 94.57 | *Wickerhamiella musiphila* CBS 10697 | NG055366 | **SDBR-CMU435** | MW542584 |  |  | | |
| 50 | FLA39.3 | 560 | 100 | *Pichia manshurica* CBS 209 | MK394164 | *Pichia manshurica* | MW602363 | 3 | 3.5 | | |
| 51 | FLA40.3 | 560 | 100 | *Pichia manshurica* CBS 209 | MK394164 | *Pichia manshurica* | MW602365 |  |  | | |
| 52 | FLA41.3 | 560 | 100 | *Pichia manshurica* CBS 209 | MK394164 | *Pichia manshurica* | MW602368 |  |  | | |
| 53 | FLA48.1 | 583 | 98.8 | *Priceomyces melissophilus* CBS 6344 | KY108946 | *Priceomyces melissophilus* | MW602739 | 1 | 1.2 | | |
| 54 | FLA42.1 | 572 | 100 | *Saccharomyces cerevisiae* CBS 5493 | NG042623 | *Saccharomyces cerevisiae* | MW602369 | 1 | 1.2 | | |
| 55 | FLA17.1 | 570 | 100 | *Saccharomycopsis fodiens* CBS 8332 | NG054782 | *Saccharomycopsis fodiens* | MW602333 | 1 | 1.2 | | |
| **Supplementary Table S2** (Continued) | | | | | | | | | | |  |
| **No.** | **Isolate** | **D1/D2 sequence (nt)** | **Similarity** | **CBS type strain** | **CBS type strain Accession no.** | **Identification results** | **Accession no.** | **No. of sample found for each species** | **% Frequency of occurrence** | | |
| 56 | FLA5.3 | 562 | 98.40 | *Suhomyces xylopsoci* CBS 6037 | NG054782 | *Suhomyces xylopsoci* | KY411894 | 3 | 3.5 | | |
| 57 | FLA44.1 | 560 | 98.57 | *Suhomyces xylopsoci* CBS 6037 | NG054782 | *Suhomyces xylopsoci* | MW602371 |  |  | | |
| 58 | FLA45.1 | 558 | 98.57 | *Suhomyces xylopsoci* CBS 6037 | NG054782 | *Suhomyces xylopsoci* | MW602373 |  |  | | |
| 59 | FLA1.3 | 553 | 99.28 | *Wickerhamiella azyma* CBS 6826 | KY106315 | *Wickerhamiella azyma* | MW602310 | 8 | 9.4 | | |
| 60 | FLA8.3 | 553 | 99.28 | *Wickerhamiella azyma* CBS 6826 | KY106315 | *Wickerhamiella azyma* | MW602321 |  |  | | |
| 61 | FLA20.1 | 553 | 99.28 | *Wickerhamiella azyma* CBS 6826 | KY106315 | *Wickerhamiella azyma* | MW602336 |  |  | | |
| 62 | FLA35.2 | 552 | 99.28 | *Wickerhamiella azyma* CBS 6826 | KY106315 | *Wickerhamiella azyma* | MW602354 |  |  | | |
| 63 | FLA36.3 | 553 | 99.28 | *Wickerhamiella azyma* CBS 6826 | KY106315 | *Wickerhamiella azyma* | MW602357 |  |  | | |
| 64 | FLA37.1 | 552 | 99.64 | *Wickerhamiella azyma* CBS 6826 | KY106315 | *Wickerhamiella azyma* | MW602358 |  |  | | |
| 65 | FLA38.2 | 553 | 99.28 | *Wickerhamiella azyma* CBS 6826 | KY106315 | *Wickerhamiella azyma* | MW602360 |  |  | | |
| 66 | FLA40.4 | 551 | 99.27 | *Wickerhamiella azyma* CBS 6826 | KY106315 | *Wickerhamiella azyma* | MW602366 |  |  | | |
| 67 | FLA39.1 | 573 | 100 | *Wickerhamomyces anomalus* CBS 5759 | MH545921 | *Wickerhamomyces anomalus* | MW602361 | 1 | 1.2 | | |
| 68 | FLA11.2 | 567 | 100 | *Wickerhamomyces ciferrii* CBS 111 | NG057172 | *Wickerhamomyces ciferrii* | MW602326 | 1 | 1.2 | | |
| 69 | FLA45.4 | 531 | 98.68 | *Yamadazyma dushanensis* CBS 13914 | NG064369 | *Yamazyma dushnensis* | MW602374 | 1 | 1.2 | | |
|  | **Phylum Basidiomycota** | | | | | | | | |  |  |
| 1 | FLA2.4 | 597 | 98.99 | *Hannaella luteola* CBS 943 | NG042362 | *Hannaella luteola* | MW602313 | 1 | 1.2 | | |
| 2 | FLA26.1 | 597 | 100 | *Hannaella pagnoccae* CBS 11142 | FJ828959 | *Hannaella pagnoccae* | MW602339 | 1 | 1.2 | | |
| 3 | FLA2.1 | 594 | 100 | *Hannaella sinensis* CBS 7225 | AF189857 | *Hannaella sinensis* | MW602311 | 1 | 1.2 | | |
| 4 | FLA50 | 605 | 100 | *Moesziomyces antarcticus* CBS 5955 | KY108571 | *Moesziomyces antarcticus* | MW602380 | 2 | 2.4 | | |
| 5 | FLA53 | 605 | 100 | *Moesziomyces antarcticus* CBS 5955 | KY108571 | *Moesziomyces antarcticus* | MW602382 |  |  | | |
| 6 | FLA31.5 | 597 | 100 | *Papiliotrema flavoscens* CBS 6475 | EF056317 | *Papiliotrema flavescens* | MW602345 | 2 | 2.4 | | |
| 7 | FLA51.1 | 597 | 100 | *Papiliotrema flavoscens* CBS 7140 | EF056318 | *Papiliotrema flavoscens* | MW602381 |  |  | | |
| 8 | FLA3.1 | 605 | 99.67 | *Pseudozyma hubeiensis* CBS 10077 | KY108956 | *Pseudozyma hubeiensis* | MW602314 | 3 | 3.5 | | |
| 9 | FLA32.3 | 605 | 100 | *Pseudozyma hubeiensis* CBS 10077 | KY108956 | *Pseudozyma hubeiensis* | MW602350 |  |  | | |
| 10 | FLA35.1 | 605 | 99.67 | *Pseudozyma hubeiensis* CBS 10077 | KY108956 | *Pseudozyma hubeiensis* | MW602353 |  |  | | |
| 11 | FLA36.1 | 568 | 99.3 | *Sporidobolus pararoseus* CBS 491 | NG067256 | *Sporidobolus pararoseus* | MW602355 | 1 | 1.2 | | |
| 12 | FLA31.7 | 572 | 100 | *Sporobolomyces bannaensis* CBS 9204 | NG068721 | *Sporobolomyces bannaensis* | MW602347 | 2 | 2.4 | | |
| 13 | FLA36.2 | 572 | 99.13 | *Sporobolomyces bannaensis* CBS 9204 | NG068721 | *Sporobolomyces bannaensis* | MW602356 |  |  | | |

**Supplementary Table S3.** Details of sequences used for phylogenetic analysis of the genus *Metschnikowia* in this study.

| Yeast species | Strains | GenBank accession number | |
| --- | --- | --- | --- |
|  |  | ITS | D1/D2 |
| *Metschnikowia arizonensis* | CBS 9064^T^ | NR172732 | KY108451 |
| *Metschnikowia colocasiae* | UWOPS 03-134.2^T^ | NR164508 | AY393869 |
| *Metschnikowia dekortorum* | CBS 9063^T^ | NR164525 | KY108468 |
| *Metschnikowia lacustris* | UWOPS 12-619.2^T^ | KF647746 | ‒ |
| *Metschnikowia similis* | CBS 9737^T^ | ‒ | KY108506 |
| *Metschnikowia aberdeeniae* | CBS 10289^T^ | ‒ | NG058334 |
| *Metschnikowia shivogae* | CBS 10292^T^ | ‒ | NG058345 |
| *Metschnikowia hawaiiensis* | CBS 7432^T^ | NR164376 | KY108472 |
| *Metschnikowia mauinuiana* | CBS 10060^T^ | NR160985 | KY108487 |
| *Metschnikowia cerradonensis* | CBS 10409^T^ | ‒ | NG058335 |
| *Metschnikowia cubensis* | CBS 10833^T^ | KY108465 | KY108466 |
| *Metschnikowia ipomoeae* | CBS 8466^T^ | NR164375 | NG060836 |
| *Metschnikowia lochheadii* | CBS 8807^T^ | NR164507 | NG058341 |
| *Metschnikowia lachancei* | CBS 9131^T^ | NR164374 | KY108483 |
| *Metschnikowia matae* | UFMG-CM-Y397^T^ | NR172730 | ‒ |
| *Metschnikowia santaceciliae* | UWOPS 01-517a1^T^ | NR172726 | ‒ |
| *Metschnikowia continentalis* | UWOPS 96-173^T^ | NR164508 | AY393869 |
| *Metschnikowia borealis* | CBS 8431^T^ | NR164506 | KY108457 |
| *Metschnikowia kamakouana* | UWOPS 04-112.5^T^ | AY796024 | ‒ |
| *Metschnikowia hibisci* | CBS 8433^T^ | NR164512 | AF034128 |
| *Metschnikowia proteae* | CBS 12552^T^ | – | JN935052 |
| *Metschnikowia kipukae* | CBS 9147^T^ | KY102171 | KY106539 |
| *Metschnikowia henanensis* | CBS 12677^T^ |  | NG064322 |
| ***Metschnikowia lannaensis*** | **SDBR-CMU426^T^** | **OM213034** | **KY640629** |
| ***Metschnikowia lannaensis*** | **SDBR-CMU427** | **MZ857157** | **MW542582** |
| *Metschnikowia miensis* | NBRC 112445^T^ | NR165863 | NG067798 |
| *Metschnikowia hawaiiana* | CBS 9146^T^ | KY102130 | NG060890 |
| *Metschnikowia orientalis* | CBS 10331^T^ | KY104201 | NG058343 |
| *Metschnikowia typographi* | MR382^T^ | ‒ | KU886295 |
| *Metschnikowia agaves* | NRRL Y-17915^T^ | – | NG055391 |
| *Metschnikowia drakensbergensis* | CBS 13649^T^ | NR164382 | – |
| *Metschnikowia saccharicola* | CBS 12575^T^ | AB697760 | AB697755 |
| *Metschnikowia lopburiensis* | DMKU-RK277^T^ | AB697761 | AB697756 |
| *Metschnikowia caudata* | CBS 13651^T^ | KM233175 | – |
| *Metschnikowia taurica* | KBP Y-6724^T^ | MW579433 | MW580941 |
| *Metschnikowia drosophilae* | CBS 8809^T^ | NR0777075 | NG058338 |
| *Metschnikowia torresii* | NRRL Y-6699^T^ | ‒ | NG055411 |
| *Metschnikowia laotica* | CBS 12961^T^ | NR164084 | JX515978 |
| *Metschnikowia fructicola* | NRRL Y-27328^T^ | – | AF360542 |
| *Metschnikowia persimmonesis* | KCTC 12991BP^T^ | MF446617 | MF446618 |
| *Metschnikowia andauensis* | HA 1657^T^ | – | AJ745110 |
| *Metschnikowia citriensis* | FL01^T^ | MF538700 | MF538699 |
| *Metschnikowia rubicola* | NRRL Y-6064^T^ | MG050888 | NG073612 |
| *Metschnikowia sinensis* | CBS 10357^T^ | KY104211 | MZ651987 |
| *Metschnikowia shanxiensis* | CBS 10359^T^ | KY104210 | NG058344 |

**Supplementary Table S3.** (continued)

| Yeast species | Strains | GenBank accession number | |
| --- | --- | --- | --- |
|  |  | ITS | D1/D2 |
| *Metschnikowia leonuri* | NRRL Y-6546^T^ | MG050887 | NG073611 |
| *Metschnikowia chrysoperlae* | CBS 9803^T^ | KY104196 | KY108461 |
| *Metschnikowia ziziphicola* | CBS 10358^T^ | KY104196 | KY108509 |
| *Metschnikowia pulcherrima* | NRRL Y-7111^T^ | NR164379 | NG075437 |
| *Metschnikowia picachoensis* | CBS 9804^T^ | – | NG067768 |
| *Metschnikowia pimensis* | CBS 9805^T^ | KY102334 | KY106693 |
| *Metschnikowia lunata* | CBS 5946^T^ | – | KY108486 |
| *Metschnikowia kunwiensis* | CBS 9676 | MK394154 | KY108482 |
| *Metschnikowia corniflorae* | NRRL Y-27750^T^ | – | AY611610 |
| *Metschnikowia koreensis* | CBS 8854^T^ | – | NG058340 |
| *Metschnikowia peoriensis* | NRRL Y-5942^T^ | MG050889 | NG064474 |
| *Metschnikowia churdharensis* | CBS 15318^T^ | – | MG821162 |
| *Metschnikowia persici* | FHL-A^T^ | KY197817 | KY197814 |
| *Metschnikowia cibodasensis* | UICC Y-335^T^ | NR138172 | NG055300 |
| *Metschnikowia reukaufii* | NRRL Y-7112^T^ | MG050896 | NG055410 |
| *Metschnikowia rancensis* | CBS 8174^T^ | NR155369 | NG055309 |
| *Metschnikowia vanudenii* | CBS 9134^T^ | KY104212 | KY108507 |
| *Metschnikowia maroccana* | NRRL Y-63972^T^ | – | NG075240 |
| *Metschnikowia chrysomelidarum* | CBS 9904^T^ | KY102031 | KY106396 |
| *Metschnikowia baotianmanensis* | NYNU 15613^T^ | Ku128712 | NG079504 |
| *Metschnikowia gelsemii* | CBS 10509^T^ | NR164510 | KY106461 |
| *Metschnikowia gruessii* | NRRL Y-17805^T^ | – | U45737 |
| *Metschnikowia viticola* | NCAIM Y.01705^T^ | OW987332 | AY626892 |
| *Metschnikowia noctiluminum* | NRRL Y-27753^T^ | DQ002495 | NG055317 |
| *Metschnikowia kofuensis* | NRRL Y-27226^T^ | DQ002493 | AF158019 |
| *Metschnikowia colchici* | CBS 12461^T^ | JX656748 | JX656747 |
| *Metschnikowia australis* | CBS 5847^T^ | – | KY108453 |
| *Metschnikowia bicuspidata* | NRRL Y-17916^T^ | – | NG055421 |
| *Metschnikowia zobellii* | CBS 4821^T^ | KY104215 | L10691 |
| *Metschnikowia anglica* | NRRL Y-7298^T^ | NR158858 | NG064475 |
| *Saccharomyces cerevisiae* | CBS 1171^T^ | NR111007 | NG042623 |
| *Eremothecium cymbalariae* | CBS 270.75^T^ | NR119481 | NG042628 |

The superscript “^T^” indicates type species. “‒” indicates the absent of data in GenBank database. Yeast strains and sequences obtained from this study are in red.

**Supplementary Table S4.** Details of sequences used for phylogenetic analysis of the genus *Wickerhamiella* in this study.

| Yeast species | Strains | GenBank accession number | |
| --- | --- | --- | --- |
|  |  | ITS | D1/D2 |
| *Wickerhamiella azyma* | CBS 6826^T^ | NR164371 | EF536346 |
| *Wickerhamiella azymoides* | UFMG 2287^T^ | NR164509 | DQ985171 |
| *Wickerhamiella parazyma* | CBS 11563^T^ | NR164511 | EF601044 |
| *Wickerhamiella nectarea* | CBS 14162^T^ | – | NG064391 |
| ***Wickerhamiella camelliae*** | **SDBR-CMU428^T^** | **MZ857161** | **KY411895** |
| ***Wickerhamiella camelliae*** | **SDBR-CMU429** | **MZ857162** | **MW542579** |
| ***Wickerhamiella camelliae*** | **SDBR-CMU430** | **MZ857163** | **MW542580** |
| ***Wickerhamiella camelliae*** | **SDBR-CMU431** | **MZ857167** | **MW542585** |
| *Wickerhamiella dulcicola* | CBS 12588^T^ | NR164520 | JQ180256 |
| *Wickerhamiella cachassae* | UFMG-D5L7^T^ | JQ780060 | JQ180255 |
| *Wickerhamiella natalensis* | CBS 14161^T^ | – | NG064390 |
| *Wickerhamiella occidentalis* | CBS 8452^T^ | – | NG057641 |
| *Wickerhamiella lipophila* | CBS 8458^T^ | – | NG058437 |
| *Wickerhamiella australiensis* | CBS 8456^T^ | – | NG058435 |
| *Wickerhamiella cacticola* | NRRL Y-27362^T^ | NR160987 | AF046035 |
| *Wickerhamiella nakhonpathomensis* | TBRC 14875^T^ | LC632019 | LC604625 |
| *Wickerhamiella pagnoccae* | CBS 12178^T^ | NR164518 | HQ593535 |
| *Wickerhamiella kiyanii* | CBS 12905^T^ | NR164522 | JX978398 |
| *Wickerhamiella jalapaonensis* | CBS 10935^T^ | – | KY106530 |
| *Wickerhamiella drosophilae* | CBS 8459^T^ | – | NG057642 |
| *Wickerhamiella vanderwaltii* | CBS 5524^T^ | NR164372 | U62313 |
| *Wickerhamiella goesii* | IMUFRJ 52102^T^ | – | JN790617 |
| *Wickerhamiella alocasiicola* | CBS 10702^T^ | KY101923 | EU284106 |
| *Wickerhamiella pararugosa* | CBS 1010^T^ | NR155986 | U62306 |
| *Wickerhamiella shivajii* | CBS 15893^T^ | NR164469 | MK606540 |
| *Wickerhamiella brachini* | NYNU 15885^T^ | NR173256 | KU128726 |
| *Wickerhamiella spandovensis* | CBS 6875^T^ | KY106776 | NR155988 |
| *Wickerhamiella sergipiensis* | UFMG-R188^T^ | – | NG059907 |
| *Wickerhamiella allomyrinae* | CBS 13167^T^ | NR160319 | NG064357 |
| *Wickerhamiella dianesei* | UWOPS 00-107.1^T^ | – | AF313369 |
| *Wickerhamiella kurtzmanii* | UWOPS 00-192.1^T^ | MF975533 | MF975533 |
| *Wickerhamiella bombiphila* | CBS 9712^T^ | – | NG055315 |
| *Wickerhamiella domercqiae* | NRRL Y-6692^T^ | DQ911462 | DQ438240 |
| *Wickerhamiella versatilis* | CBS 1752^T^ | NR151812 | KY106869 |
| *Wickerhamiella galacta* | NRRL Y-17645^T^ | – | DQ438239 |
| ***Wickerhamiella thailandensis*** | **SDBR-CMU432^T^** | **MZ857160** | **KY411893** |
| ***Wickerhamiella thailandensis*** | **SDBR-CMU433** | **MZ857164** | **MW542581** |
| ***Wickerhamiella thailandensis*** | **SDBR-CMU434** | **MZ857165** | **KY640634** |
| ***Wickerhamiella thailandensis*** | **SDBR-CMU435** | **MZ857166** | **MW542584** |
| *Wickerhamiella musiphila* | CGMCC 2.3479^T^ | NR137659 | EU284104 |
| *Wickerhamiella sorbophila* | NRRL Y-7921^T^ | DQ911460 | DQ438229 |
| *Wickerhamiella infanticola* | NRRL Y-17858^T^ | DQ911458 | DQ438230 |
| *Wickerhamiella tropicalis* | DMKU VGT1-19^T^ | NR172735 | MH142264 |
| *Wickerhamiella verensis* | CECT 12028^T^ | NR169892 | EF141077 |
| *Wickerhamiella osmotolerans* | DMKU VGT1-14^T^ | MN194615 | MH141490 |

**Supplementary Table S4. (continued)**

| Yeast species | Strains | GenBank accession number | |
| --- | --- | --- | --- |
|  |  | ITS | D1/D2 |
| *Wickerhamiella siamensis* | DMKU-SE106^T^ | NR155951 | NG058438 |
| *Wickerhamiella qilinesis* | NYNU 146103^T^ | NR173251 | NG079498 |
| *Wickerhamiella pterostichi* | NYNU 15896^T^ | NR173257 | KU128723 |
| *Wickerhamiella kazuoi* | NBRC 102565^T^ | – | NG055687 |
| *Wickerhamiella fructicola* | CBS 12902^T^ | NR158998 | NG059950 |
| *Wickerhamiella hasegawae* | CBS 12089^T^ | KY102129 | AB306510 |
| *Wickerhamiella slavikovae* | CBS 12417^T^ | – | NG055371 |
| *Saccharomyces cerevisiae* | CBS 1171^T^ | NR111007 | NG042623 |
| *Eremothecium cymbalariae* | CBS 270.75^T^ | NR119481 | NG042628 |

The superscript “^T^” indicates type species. “‒” indicates the absent of data in GenBank database. Yeast strains and sequences obtained from this study are in red.
